# Latroeggtoxin-VI protects nerve cells and prevents depression by inhibiting NF-κB signaling pathway activation and excessive inflammation

**DOI:** 10.3389/fimmu.2023.1171351

**Published:** 2023-05-15

**Authors:** Haiyan Wang, Yiwen Zhai, Zhixiang Lei, Si Chen, Minglu Sun, Panfeng Yin, Zhigui Duan, Xianchun Wang

**Affiliations:** State Key Laboratory of Developmental Biology of Freshwater Fish, Protein Chemistry Laboratory, College of Life Sciences, Hunan Normal University, Changsha, Hunan, China

**Keywords:** latroeggtoxin-VI, neuroprotection, anti-depression, RAW264.7 cell, PC12 cell, depression mouse model, anti-inflammation

## Abstract

Depression has a high incidence and seriously endangers human health. Accumulated evidence indicates that targeting neuroinflammation is a potential avenue for neuroprotection and thus depression prevention. Herein, the effects of latroeggtoxin-VI (LETX-VI), a bioactive protein from the eggs of spider *Latrodectus tredecimguttatus*, on lipopolysaccharide (LPS)-induced inflammation and depression were systematically investigated using RAW264.7 macrophages and depression mouse model. Pretreatment with LETX-VI suppressed LPS-evoked NF-κB signaling pathway activation, inhibited LPS-induced over-production of NO, iNOS, IL-6 and TNF-α; at the same time LETX-VI mitigated the inhibitory effect of LPS on the expression of anti-inflammatory factors such as Arg-1, thereby suppressing oxidative stress and excessive inflammation. Culture of PC12 cells with the conditioned medium of RAW264.7 cells pretreated with LETX-VI demonstrated the neuroprotective effect of LETX-VI due to its anti-inflammation effect. In the LPS-induced depression mouse model, pretreatment with LETX-VI improved the LPS-induced depression-like behaviors, inhibited the activation of microglia and astrocytes, prevented the down-regulation of Nurr1 expression and alleviated the LPS-caused adverse changes in the brain tissues. Taken together, these *in vitro* and *in vivo* findings provide powerful insights into the anti-inflammation-based neuroprotective and antidepressant mechanisms of LETX-VI, which is helpful to deeply reveal the biological effects and potential applications of LETX-VI.

## 1 Introduction

Adverse environment usually makes people produce stress, anxiety and insecurity, which often lead to many psychological and mental diseases, such as depression that is one of the most common mental diseases in the world. Patients with depression not only have abnormal brain function, but also have certain changes in brain structure, such as the loss of neuron volume and the reduction of neuron number (1). In many cases, inflammation is the body’s response to stress and a large number of literatures have reported that neuroinflammation is related to the pathogenesis of depression and many neurodegenerative diseases (2–7). The main feature of brain inflammation is the activation of astrocytes and particularly microglia, the resident macrophages of the central nervous system (8, 9). The activated microglia release pro-inflammatory cytokines and free radicals that initiate or amplify the neuronal damage response, which then activates other microglia and thus form a cyclic mechanism of action until the pathogen is eliminated (10–12). Patients with depression were found to have increased plasma concentrations of pro-inflammatory cytokines, including TNF-α, IL-1β, IL-6, etc (13–15).

Depressive-like behaviors in animals could be induced by cytokines or cytokine inducers such as lipopolysaccharide (LPS) administration or chronic mild stress (16–19). In the relevant researches, LPS was commonly employed to induce inflammatory responses in macrophages, which was used as a classical model to evaluate the anti-inflammatory activity of active substances (20–22). On the other hand, anti-inflammatory agents were used to suppress pro-inflammatory cytokine level in both humans and animals (23, 24). The accumulated evidence has suggested that activation of microglia-induced neuroinflammation plays a crucial role in the pathophysiology of depression; targeting the neuroinflammation by inhibiting microglia/macrophage activation is considered a possible strategy to reduce neuroinflammation, protect nerve cells and alleviate depression (25–29). Therefore, deepening the systematic investigation on the relationship between inflammation and both of nerve cell injury and depression, and screening the bioactive molecules with a strong potential application in anti-inflammation are of both theoretical and clinical significance.

Latroeggtoxin-VI (LETX-VI), a bioactive protein with an average molecular weight of 6199, was discovered from the eggs of spider *Latrodectus tredecimguttatus*. Previous study demonstrated that LETX-VI could enter PC12 cells and promote the synthesis and release of dopamine (30–32). Dopamine plays important roles in a series of physiological functions such as movement, cognition, mood and reward. Many human disorders such as depression and Parkinson’s disease (PD) are due, in part, to dysfunctional dopaminergic systems. A series of the symptoms seen in depression, such as anhedonia and amotivation, have been more consistently associated with dysfunctions in the dopamine system (33, 34). Inflammation can decrease dopamine synthesis, packaging and release, thereby affecting the basal ganglia and dopamine to mediate depressive symptoms, and sabotaging and/or circumventing the efficacy of standard antidepressant treatments (35). The close relationship among dopamine, inflammation, neuron injury and depression aroused our great interest in probing into the possible actions of LETX-VI in the relevant aspects. The results of our present study demonstrated that LETX-VI could exert neuroprotective and antidepressive effects by inhibiting NF-κB signaling pathway and suppressing excessive inflammation.

## 2 Materials and methods

### 2.1 Preparation of recombinant LETX-VI

Recombinant LETX-VI was prepared through heterologous expression in *E. coli* based on the method described (31). In brief, the total RNA extracted from *L. tredecimguttatus* eggs was used as the template for synthesis of the first cDNA strand. Nested PCR was employed to amplify the LETX-VI gene, which was cloned into expression vector pET32a, followed by transforming into *E. coli* BL21 (DE3) to express the fusion protein by IPTG induction. The expressed fusion protein was affinity purified with Ni-NTA beads, digested with enterokinase, separated by RP‐HPLC and confirmed with mass spectrometry.

### 2.2 Cell culture and determination of the cytotoxicity of LETX-VI and LPS toward RAW264.7 cells

RAW 264.7 cells were purchased from Cell Bank of Chinese Academy of Sciences (Shanghai, China) and seeded in the DMEM medium containing 15% fetal bovine serum (FBS), 100 U/ml of penicillin and 100 µg/ml of streptomycin, followed by incubation in a humidified incubator at 37 °C and 5% CO_2_. The culture medium was replaced every 1-2 days and the cells at the exponential growth phase were adopted for drug treatment, followed by the determnations of cytotoxicity, LDH, NO, IL-6, TNF-α, etc.

To detect the possible cytotoxicity of LETX-VI and LPS toward RAW264.7 cells, the cells were seeded in a 96-well plate and incubated overnight and then the wells were randomly grouped into blank, control and test groups, each containing three wells. After the cells pasted wall, the culture medium was gently removed by aspiration. LETX-VI at different concentrations (0.1, 1, 4 and 8 μM) prepared with FBS-free DMEM were separately added into the three wells of test group, 100 μl each. When LPS was added, the last concentrations were 0.01, 0.1, 1 and 10 μg/ml. The blank group received no cell seeding and the control group received no drugs. After 24 h culture, 10 μl CCK-8 solution (AbMole BioScience, Shanghai, China) was added into each well, followed by incubation at 37 °C for 3 h. The cell viability was determined by measuring the optical density (OD) absorbance of each well at the wavelength of 450 nm with a microplate reader (Bio-Rad Laboratories, CA, USA). In addition, in order to detect the effect of combined application of LETX-VI and LPS on the viability of RAW264.7 cells, LETX-VI up to 10 μM was first applied to the cells for 12 h and then 1 μg/ml LPS was added to treat the cells for another 12 h.

The activity of lactate dehydrogenase (LDH) released from RAW264.7 cells into culture medium after LETX-VI treatment was also used as an index for evaluating the cytotoxiciy of LETX-VI toward RAW264.7 cells. The activity determination was performed using a LDH Cytotoxicity Assay Kit (Beyotime Biotechnology Co., Ltd., Shanghai, China) according to the instructions of the manufacturer. In brief, RAW264.7 cells were seeded in a 96-well plate and the wells were divided into 4 groups: blank group, control group, maximum LDH activity group, and LETX-VI treatment group. After the cells were allowed to grow to approximately 80-90% confluence, the culture medium was removed and LETX-VI prepared with FBS-free DMEM to different concentrations (0.15, 0.3, 0.6, 1.3, 2.5, 5 and 10 μM) were added into the wells in LETX-VI treatment group, each concentration in quintuplicate. The cell lysis buffer was added into the cells in maximum LDH activity group. The cells in blank group received no cells and those in control group received no LETX-VI. After incubation for 24 h, the culture medium was collected for determining the activity of released LDH.

### 2.3 Assay of NO released from RAW264.7 cells

The nitric oxide (NO) released from RAW264.7 cells into the culture medium was quantitatively determined using a Nitric Oxide (NO) Content Assay Kit (Beyotime, Nanjing, China) according to the instructions of the manufacturer. For detecting the influence of LPS at different concentrations on NO level, LPS at 0, 0.01, 0.1, 1 and 10 µg/ml was used to treat RAW264.7 cells for 24 h. When the anti-inflammatory effect of LETX-VI was investigated, different concentrations of LETX-VI (0.1, 1 and 4 µM) were used to pretreat the RAW264.7 cells for 12 h and then LPS (1 µg/ml) was used to treat the cells for another 12 h. After the RAW264.7 cells were treated with or without various concentrations of LETX-VI and/or LPS, the culture medium was separated from the cells by centrifugation. 50 µl of cell-free culture culture was mixed with 50 µl of Griess reagent I and 50 µl of Griess reagent II, and the absorbance was recorded at 540 nm using a microplate reader (Bio-Rad Laboratories, CA, USA). Before the NO determination, a standard sodium nitrite curve was established and the NO content was calculated according to the standard curve.

### 2.4 ELISA analyses of IL-6 and TNF-α

After treatment of the cultured RAW264.7 cells with LETX-VI and/or LPS, the levels of interleukin-6 (IL-6) and tumor necrosis factor (TNF-α) released into the culture medium were determined with ELISA kits according to the manufacturer’s instructions. The cells were randomly divided into control group, LPS treatment group and LETX-VI pretreatment group. The cells in LPS treatment group were treated with LPS (1 μg/ml) for 12 h, and those in LETX-VI pretreatment group were pretreated with LETX-VI (0.1, 1 and 4 μM) for 12 h and then were treated with LPS (1 μg/ml) for another 12 h. After the RAW264.7 cells were treated with different concentrations of LETX-VI and/or LPS, the collected and properly diluted cell-free culture media as well as the different concentrations of standard sample in a volume of 100 μl were added into 96-well ELISA plates, followed by incubation for 2 h. The solution in the wells was removed and washing buffer was used to rinse the wells 5 times. 100 μl biotinylated anti- IL-6 or TNF-α antibody was added and incubated for 1 h. After washing 5 times, 100 μl horseradish peroxidase-conjugated avidin solution was added and incubated for 20 min in the dark at room temperature, followed by extensive washing and addition of 100 μl chromogenic substrate 3,3’,5,5’-Tetramethylbenzidine (TMB). After the reaction was allowed to proceed for 20 min and then stopped using 50 μl stopping solution, the absorbance value was recorded at 450 nm with a microplate reader (Bio-Rad Laboratories, CA, USA). The concentrations of IL-6 and TNF-α were calculated according to the standard curve prepared with known concentrations of the cytokines.

### 2.5 Western blot analysis

The levels of multiple inflammation- and neuroprotection-related proteins, including inductible nitric oxide synthase (iNOS), arginase-1 (Arg-1), nuclear receptor-related 1 (Nurr1), etc, were detected with western blot analysis. The protein sample was resolved on a 12% SDS-PAGE gel in principle as described by Laemmli (36). The gel strip containing the protein of interest was cut off and the protein was transferred onto a polyvinylidene difluoride (PVDF) membrane (PALL Corporation, USA) with 100 mA for 2.5 h using a blot electrotransfer apparatus in the wet transfer method. After the PVDF membrane was blocked by treatment in 5% milk prepared in TBST buffer (50 mM Tris-HCl, 150 mM NaCl, 0.1% Tween-20, pH7.5) for 1.5 h at room temperature, the protein of interest on the membrane was first probed into with primary antibody, and then with the secondary antibody conjugated with horseradish peroxidase. The blot was visualized using the enhanced chemiluminescence (ECL) method and recorded with the ChemiDoc XRS imaging system (Bio-Rad, USA).

### 2.6 Development of LPS-induced depression mouse model and detection of anti-depressive effects of LETX-VI

The male C57BL/6J mice were purchased from Slac & Jingda Corporation of laboratory animals, Changsha, China. Ethical guidelines for the use of laboratory animals allowed experiments to be conducted on mice, and the experiments were approved by the Ethics Committee of Hunan Normal University. Development of LPS-induced depression mouse model was performed by according to the previous methods (37, 38). The male C57BL/6J mice were randomly divided into control, LPS treatment and LETX-VI/LPS combined treatment groups, 10 mice in each group. The control mice were intraperitoneally received physiological saline, the mice in LPS treatment group mice were intraperitoneally received LPS (0.83 mg/kg), and those in LETX-VI/LPS combined treatment group were intraperitoneally injected with LETX-VI (3 mg/kg body weight), 12 h later injected with LETX-VI again, and 1 h later injected with LPS (0.83 mg/kg) (17, 19, 25, 39, 40). Twenty-four h after LPS injection, the behavioral tests, including sucrose preference test, tail suspension test and forced swimming test, were performed. The levels of multiple proteins that are related with inflammation and neuroprotection were detected with the methods including western blot analysis, immunofluorescent staining, etc.

#### 2.6.1 Sucrose preference test

Sucrose preference test was performed according to the previously described method (41, 42). Briefly, the mice were housed separately and independently, freely accessing to right amount of food and 1% sucrose solution in two bottles for 24 h; then the sucrose solution in one bottle was replaced with water for another 24 h. The positions of the bottles were exchanged every 12 h to avoid position bias. After the two-day adaptation period, the mice were fasted from the food as well as water and sucrose solution for 24 h. After drug treatment, the mice were allowed to freely access to the two bottles and right amount of food. The weights of the two bottles were weighted in advance and 24 h later, the bottles were taken out and weighed to determine sucrose solution and water consumption. Sucrose preference (%) = (sucrose bottle weight (g)/sucrose bottle weight (g)) + water bottle weight (g)) × 100.

#### 2.6.2 Tail suspension test

The tail suspension test was carried out based on the method described by Steru et al. (43). The mouse was suspended with its head down by using an adhesive tape to stick the tail to a horizontal stick (about 30 cm from the ground) at 1-2 cm from the tail tip. The test was performed in an opaque box (30×30×25 cm) with a square opening (15×15 cm) for easy observation. In the box, the mouse was acoustically and visually isolated, being more than 15 cm from the nearest object. At the beginning, the mouse struggled to overcome the abnormal position, but after a period of time remained movement less thereby showing ‘stillness’, which was observed for 6 min and the stillness time within last 4 min was recorded.

#### 2.6.3 Forced swimming test

Forced swimming test (FST) was made according to the previously described method with minor modifications (44). The mouse was hold by grabbing its tail and gently put into a glass cylinder (21 cm in height and 14 cm in diameter) containing 23-25 °C water with a depth of about 10 cm, preventing the animal’s head from being submerged in the water. The mouse was allowed to swim for 6 min and the stillness time within last 4 min was recorded, including the time for floating in water, not struggling and raising its head above the water. After test completion, the dirty water in cylinder was replaced with fresh water, and the mouse was wiped, dried with warm air and put back to the cage.

### 2.7 Immunofluorescent staining

Immunofluorescent staining of the proteins of interest in mouse brain tissues, including ionized calcium binding adaptor molecule-1 (Iba-1), glial fibrillary acidic protein (GFAP), nuclear receptor related protein 1 (Nurr1), and tyrosine hydroxylase (TH), was carried out according to the previous methods with some modifications (45, 46). After the mice were executed by dislocation of cervical vertebra following CO_2_ anesthesia, their brain was quickly dissected and fixed with 4% paraformaldehyde for 24 h at 4 °C. The tissues at different locations of the brain were sampled and washed three times with PBS buffer (pH 7.4). The samples were put into a dehydration box and underwent the gradient alcohol dehydration with 75-100% alcohol, followed by treatment with alcohol-benzene mixture, dimethylbenzene and 65 °C melted paraffin. Then the samples were embedded into paraffin and sliced into sections using a paraffin slicer. After the sections were dewaxed in dimethylbenzene, rehydrated in the decreasing concentrations of ethanol and washed in distilled H_2_O, antigenretrieval with microwave heating and blockage in 3% BSA were performed. Then the section was incubated with the primary antibody diluted in PBS at 4 °C overnight. After washing three times with the PBS buffer, the section was incubated with a fluoresine-conjugated secondary antibody at room temperature in dark. The nuclei were stained with 2-(4-Amidinophenyl)-6-indolecarbamidine dihydrochloride (DAPI). Immunofluorescent images were recorded with a fluorescence microscope (Nikon Eclipse C1, Japan).

### 2.8 Nissl staining

Nissl staining was performed according to the described method with some modifications (47, 48). Briefly, the sections of brain tissues from mice were sequentially treated with dimethylbenzene, anhydrous ethanol, 75% ethanol and distilled H_2_O. The sections were stained in toluidine blue for 5 min, washed with distilled H_2_O and treated with Nissl differentiation solution. After being washed with distilled H_2_O, the sections were dried in an oven and treated with dimethylbenzene for 10 min, followed by sealing with neutral gum and observation under a light microscope (Nikon EclipseE100, Japan).

### 2.9 Assay of hemolysis and blood biochemical indexes

The possible hemolytic activity of LETX-VI was assayed using the modified method of Evans et al. (49). After male C57BL/6J mice were anaesthetized with ether, the eyeballs of mice were removed and the blood was collected into EDTA-K_2_ (2 mg/ml blood)-coated tubes to prevent coagulation, followed by refrigerated centrifugation at 800 g for 5 min to remove plasma. The obtained red blood cells were washed with isotonic PBS buffer four times. The cells were used in hemolysis assay at a final concentration of 1% (vol/vol). For negative control, isotonic PBS buffer was added; for positive control, 0.1% Triton X-100 was added. LETX-VI was diluted in a two-fold series dilution with PBS, ranging from 250 to 1.95 µM. Assays were incubated at 37°C for 30 min and then centrifuged at 12 000 g for 3 min at 4°C to remove unlysed red blood cells. An aliquot of the supernatants containing released hemoglobin was transferred to the wells of a 96-well plate, and the A490 was measured. Hemolytic activity (%) was calculated as follows:


$$({\text{A}}_{\text{t}\text{e}\text{s}\text{t}\;\text{s}\text{a}\text{m}\text{p}\text{l}\text{e}}-{\text{A}}_{\text{n}\text{e}\text{g}\text{a}\text{t}\text{i}\text{v}\text{e}\;\text{c}\text{o}\text{n}\text{t}\text{r}\text{o}\text{l}}/{\text{A}}_{\text{p}\text{o}\text{s}\text{i}\text{t}\text{i}\text{v}\text{e}\;\text{c}\text{o}\text{n}\text{t}\text{r}\text{o}\text{l}}-{\text{A}}_{\text{n}\text{e}\text{g}\text{a}\text{t}\text{i}\text{v}\text{e}\;\text{c}\text{o}\text{n}\text{t}\text{r}\text{o}\text{l}})\times 100$$

For determining the effects of LETX-VI on the blood biochemical indexes of mice, sixteen male C57BL/6J mice were divided into one control group and three test groups, each with four mice. The blood was collected at 30, 60 and 90 min after intraperitoneal injection of LETX-VI at a concentration of 3 mg/kg body weight, and the plasma was obtained after centrifugation. The control mice were injected with sterile physiological saline. The selected blood biochemical indexes, including those closely related with the functions of liver and kidney such as albumin, glutamate pyruvate transaminase, creatinine, were determined with an automatic biochemical analyzer (DXC800, Beckman, USA).

### 2.10 Statistical analysis

All the experiments were conducted at least in triplicate. The data were displayed as mean ± standard deviation of replicated measurements. The statistical analyses were performed using GraphPad software version 5.0 (GraphPad Software, Inc. La Jolla, CA, USA). One-way ANOVA with Tukey’s *post hoc* test was used to analyze differences among three or more groups, and a two-tailed Student’s t-test was used to analyze the differences between 2 groups. The difference was considered significant at *P*< 0.05.

## 3 Results

### 3.1 Cytotoxicity of LETX-VI and LPS toward RAW264.7 cells

**Figure 1:**
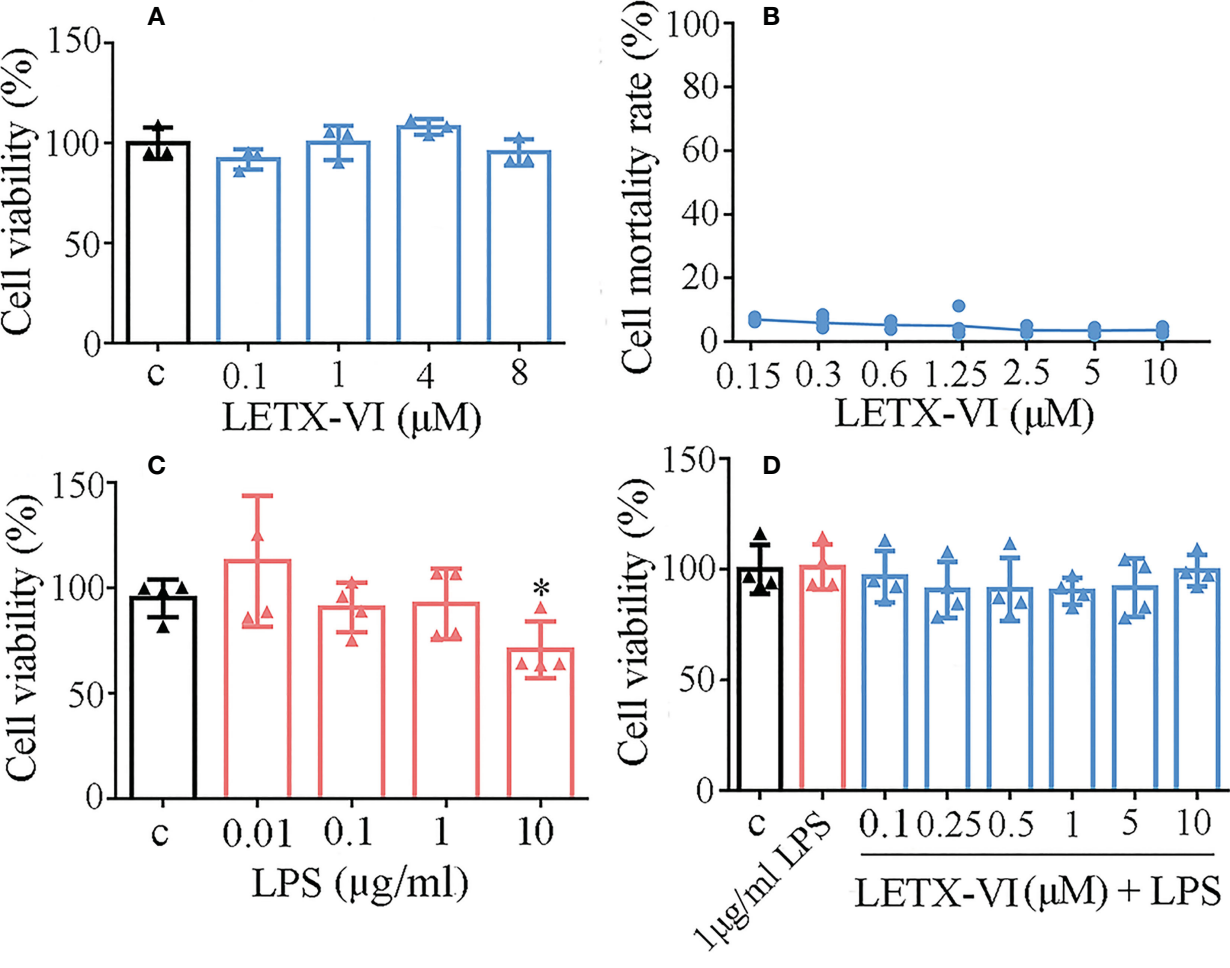
Cytotoxicity analysis of LETX-VI and LPS toward RAW264.7 cells. **(A)** Effect of LETX-VI on the viability of RAW264.7 cells. **(B)** Mortality rate of RAW264.7 cells after treatment with different concentrations of LETXI-VI, measured with lactate dehydrogenase determination. **(C)** Effect of LPS on the viability of RAW264.7 cells. *P< 0.05 vs C (control). **(D)** Effect of LETX-VI combined with LPS on the viability of RAW264.7 cells. n ≥ 3.

Before the effects of LETX-VI on LPS-induced inflammation of RAW264.7 cells were investigated, the potential cytotoxicity of LETX-VI and LPS toward the macrophages was detected to screen the optimal experimental concentrations. As shown in **Figure 1A**, LETX-VI up to 8 μM did not obviously influence the viability of RAW264.7cells after treatment for 24 h, suggesting that the cytotoxicity of LETX-VI against the macrophages, if present, is limited within this concentration range, which was supported by the results of released lactate dehydrogenase determination that LETX-VI up to 10 μM caused no obvious changes in the mortality rate of RAW264.7 cells (**Figure 1B**). Treatment of the RAW264.7 cells with LPS at concentrations of 0.01, 0.1 and 1 μg/ml for 24 h has no significant adverse effect on the viability of the cells, although LPS at 10 μg/ml led to a significant decrease in the cell viability, compared with the control (*P*< 0.05) (**Figure 1C**). Furthermore, when LETX-VI up to 10 μM was used to pretreat the macrophages before 1 μg/ml LPS application, the viability of macrophages was not significantly changed (**Figure 1D**). These findings demonstrate that LETX-VI up to 10 μM and LPS up to 1 μg/ml as well as their combined application were not obviously cytotoxic toward RAW264.7 cells.

### 3.2 Inhibitory effects of LETX-VI on LPS-induced production of pro-inflammation factors

#### 3.2.1 Appropriate concentration of LPS to induce pro-inflammation factor production

**Figure 2:**
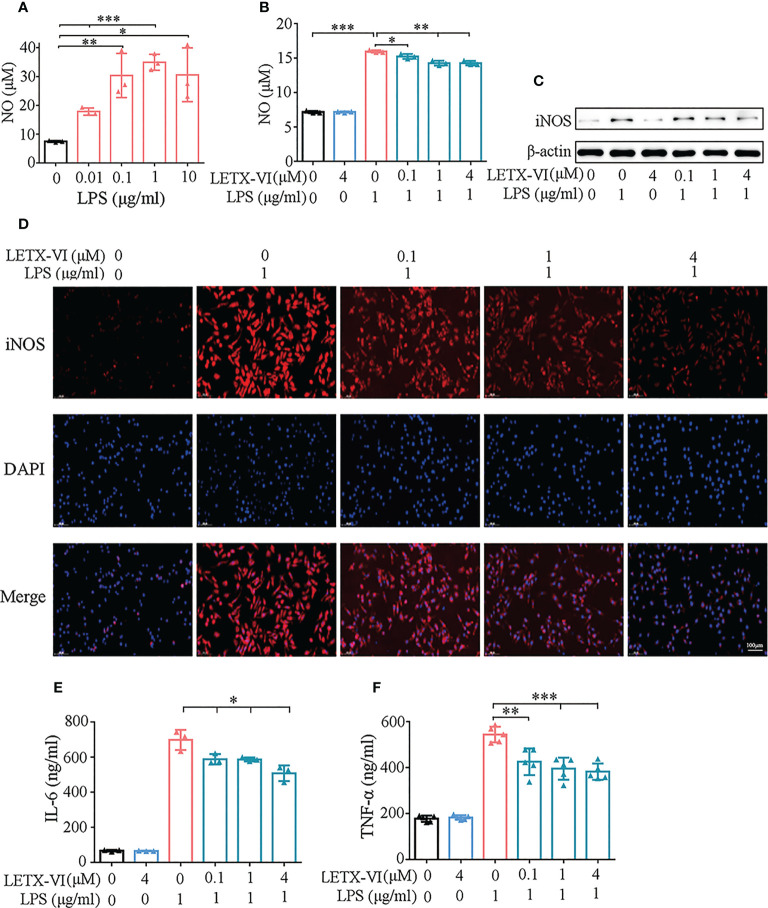
Detection of the inhibitory effects of LETX-VI on LPS-induced Pro-inflammation factor production by RAW 264.7 cells. **(A)** Screening of the appropriate concentration for LPS to induce pro-inflammation factor production. **(B)** Effects of LPS and LETX-VI on NO level. **(C)** Effects of LPS and LETX-VI on iNOS level. Gray analysis was shown in **Supplementary Figure 1A**. **(D)** Analysis of the effects of LPS and LETX-VI on iNOS level by laser confocal scanning microscopy. Relative fluorescence intensity was shown in **Supplementary Figure 1B**. **(E)** Effects of LPS and LETX-VI on IL-6 level. **(F)** Effects of LPS and LETX-VI on TNF-α level. *P< 0.05; **P< 0.01, ***P< 0.001. n ≥ 3.

For screening the appropriate experimental concentration of LPS to induce the inflammation of RAW264.7 cells, different concentrations (0.01, 0.1, 1 and 10 μg/ml) of LPS were used to treat RAW264.7 cells for 24 h and took the concentration of released NO as the detection index of inflammation, considering that NO is a free radical playing an important role in inflammation. The resulting data showed the amount of released NO was highest when 1μg/mL LPS was applied (**Figure 2A**). Moreover, LPS at this concentration did not obviously influence the viability of the RAW264.7 cells (**Figure 1C**). Therefore, 1μg/ml LPS was used in the following experiments to induce the inflammation of RAW264.7 cells, although there may be some differences among the LPS concentrations needed to induce different inflammatory factors from the RAW264.7 cells.

#### 3.2.2 LETX-VI decreases LPS-induced NO production

Treatment of RAW264.7 cells with 4 μM LETX-VI alone for 12 h showed no obvious influence on the NO content in culture medium and, however, 1μg/ml LPS treatment resulted in an increase in the NO level (*P*<0.001). Pretreatment with LETX-VI (0.1, 1 and 4 μM) before LPS application showed a significant inhibitory effect on LPS-induced increase in the NO production, with *P<* 0.05 and 0.01, respectively (**Figure 2B**).

#### 3.2.3 LETX-VI abates LPS-induced upregulation of iNOS

Western blot analysis demonstrated that 1μg/ml LPS treatment significantly increased the abundance level of this enzyme in RAW264.7 cells, while 4 μM LETX-VI alone displayed no obvious effect. Pretreatment of the cells with LETX-VI (0.1, 1 and 4 μM) before LPS application abated LPS-induced up-regulation of this enzyme (**Figure 2C**; **Supplementary Figure 1A**). For further confirming the effect of LETX-VI on iNOS level, we employed immunofluorescent staining to detect the iNOS level in RAW264.7 cells. Laser confocal scanning microscopy showed that LPS treatment significantly increased the fluorescence intensity of iNOS compared with the control and, when LETX-VI at 0.1, 1 and 4 μM were employed to pretreat the RAW264.7 cells, the fluorescence intensity was decreased as the LETX-VI concentration increased, with the fluorescence intensity being comparable to that of the control when LETX-VI concentration of LETX-VI was 4 μM (**Figure 2D**; **Supplementary Figure 1B**), which supported the conclusion drawn by western blot analysis (**Figure 2C**; **Supplementary Figure 1A**).

#### 3.2.4 LETX-VI reduces LPS-induced IL-6 production

After treating RAW264.7 cells with 1 μg/ml LPS in the same manner described above, ELISA analysis indicated that LPS treatment significantly increased the level of IL-6, and 4 μM LETX-VI alone did not obviously affect that of IL-6 compared with the control. However, LETX-VI pretreatment significantly reduced IL-6 production caused by LPS stimulation (*P*<0.05) (**Figure 2E**).

#### 3.2.5 LETX-VI decreases LPS-induced TNF-α production

When the effects of LPS and LETX-VI on TNF-α were investigated, the experimental results showed that treatment of the RAW264.7 cells with 1μg/ml LPS remarkably increased the level of TNF-α and 4 μM LETX-VI itself showed no obvious influence; however pretreatment with different concentrations (0.1, 1 and 4 μM) of LETX-VI significantly decreased the rising amplitude of TNF-α induced by LPS (*P<* 0.01 and 0.001, respectively) (**Figure 2F**).

All the observations demonstrate that, under the present experimental conditions, LETX-VI itself has no obvious effects on the level of pro-inflammation factors; however, pretreatment with LETX-VI attenuates or inhibits LPS-induced production of pro-inflammation factors.

### 3.3 LETX-VI abates LPS-induced inflammation by suppressing NF-κB signaling pathway activation caused by LPS

**Figure 3:**
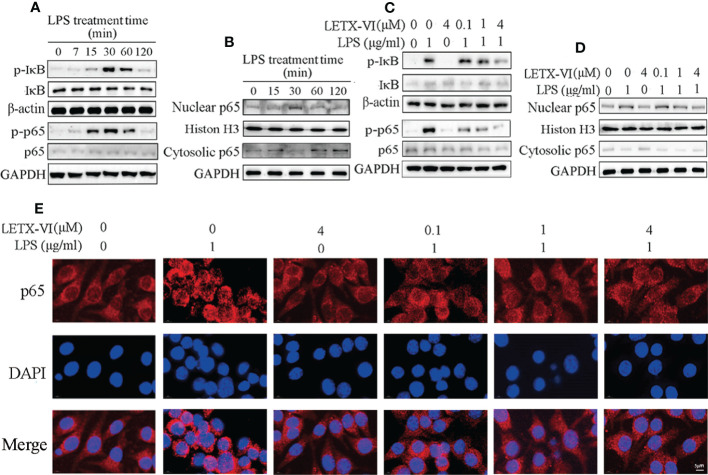
LETX-VI abates LPS-induced inflammation of RAW264.7 cells by suppressing NF-κB signaling pathway activation caused by LPS. **(A)** Treatment of RAW264.7 cells with 1 µg/ml LPS for 30 min or so promoted the phosphorylation of IκB and S536 in p65, leading to increased phosphorylated IκB (p-IκB) and S536 in p65 (p-p65). Gray analysis was shown in **Supplementary Figures 2A**, **B**. **(B)** LPS treatment promoted the entry of p65 into the nucleus from cytosol of RAW264.7 cells. Gray analysis was shown in **Supplementary Figure 2C**. **(C)** LETX-VI pretreatment inhibited the phosphorylation of IκB and S536 in p65 induced by LPS treatment for 30 min. Gray analysis was shown in **Supplementary Figures 2D**, ****E**** **(D)** LETX-VI pretreatment inhibited the entry of p65 into nucleus induced by LPS treatment for 30 min. Gray analysis was shown in **Supplementary Figure 2F**. **(E)** Immunofluorescent staining further confirmed that LETX-VI pretreatment inhibited the entry of p65 into nucleus induced by LPS treatment for 30 min. Nuclear/Cytosolic fluorescence ratio was shown in **Supplementary Figure 2G**. n ≥ 3.

For further revealing the molecular mechanism of LETX-VI to attenuate LPS-induced inflammation of RAW264.7 cells, we experimentally observed the effects of LPS and LETX-VI on the NF-κB signaling pathway, which is a typical pathway involved in immune and inflammation (50). Nuclear transcription factor-κB (NF-κB) is an important transcription regulator and usually exists in cytosol in an inactive form of p65-p50 heterodimer associated with inhibitory protein IκB. When the IκB is activated by phosphorylation, the p65-p50 dissociates from IκB and enters the nucleus to regulate transcription of multiple inflammatory factors (50, 51). Our experimental results showed that treatment of RAW264.7 cells with 1 µg/ml LPS for 30 min or so promoted the phosphorylation of IκB and the serine residue 536 (S536) in p65, with the highest promoting efficiency at 30 min (**Figure 3A**; **Supplementary Figures 2A, B**), which is in favorable for p65-p50 heterodimer to dissociate from IκB and enter the nucleus from the cytosol. After we separated the nucleus from cytosol of RAW264.7 cells and detected the distribution of p65 in these two subcellular factions, we found that, after LPS treatment for 30 min, the p65 level in the nucleus was increased and that in cytosol was accordingly decreased (**Figure 3B**; **Supplementary Figure 2C**), which demonstrated that LPS treatment of RAW264.7 cells indeed promoted the entry of P65 into the nucleus and activated the NF-κB signaling pathway.

When 4 µM LETX-VI was used alone to treat RAW264.7 cells, the phosphorylation levels of IκB and S536 in p65 were not obviously affected. However, LETX-VI (0.1, 1 and 4 µM) pretreatment for 12 h before LPS treatment for 30 min attenuated or reversed LPS-induced increase in the phosphorylation of IκB and S536 in p65 (**Figure 3C**; **Supplementary Figures 2D, E**), which is unfavorable to the entry of p65 into the nucleus. For verifying the effect of LETX-VI pretreatment on the p65 entry into the nucleus, the nucleus and cytosol were separated and their p65 content was measured. The results showed that, after 1 μg/ml LPS treatment for 30 min, the p65 level in nucleus was increased and that in cytosol was accordingly decreased; application of 4 µM LETX-VI alone did not influence the distribution of p65 between the nucleus and cytosol. However, pretretment of the RAW264.7 cells with LETX-VI (0.1, 1 and 4 µM) before LPS treatment attenuated or abolished the LPS-evoked increase in the nuclear p65 level (**Figure 3D**; **Supplementary Figure 2F**). In order to further confirm the effects of LPS and LETX-VI on the entry of p65 into the nucleus, immunofluorescent staining was employed to monitor the changes in the subcellular distribution of p65. Laser confocal scanning microscopic observation indicated that LPS induced an obvious increase in p65 fluorescence intensity near and in the nucleus, suggesting that LPS promoted p65 to move to and enter the nucleus. LETX-VI pretreatment attenuated the LPS-caused increase in the fluorescence intensity of nuclear p65 (**Figure 3E**; **Supplementary Figure 2G**), supporting the conclusion drawn with western blot analysis (**Figure 3D**; **Supplementary Figure 2F**). These results indicate that under the present experimental conditions LPS treatment activated the NF-κB signaling pathway and promoted the entry of p65 into the nucleus of RAW264.7 cells to increase the transcription of the genes for pro-inflammatory factors, whereas LETX-VI pretreatment could suppress the activation action of LPS on the NF-κB signaling pathway.

### 3.4 Effects of LPS and LETX-VI on the levels of M2 macrophage markers

**Figure 4:**
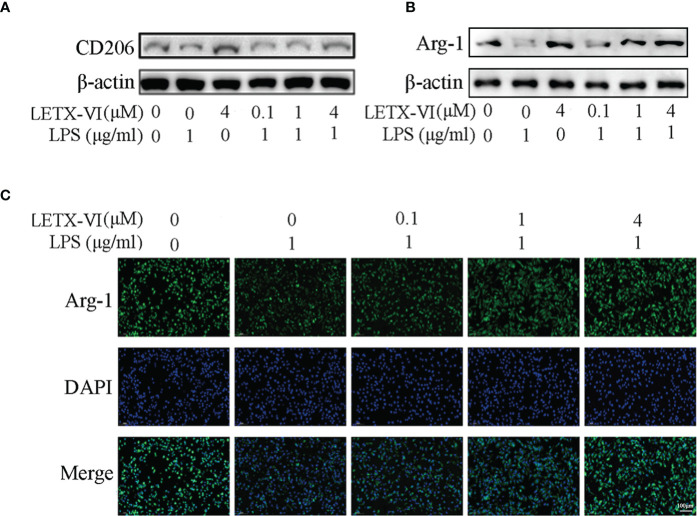
Effects of LPS and LETX-VI on the levels of CD206 and Arg-1 in RAW264.7 cells. **(A)** Effects of LPS and LETX-VI on CD206 level, detected by western blot analysis. Gray analysis was shown in **Supplementary Figure 3A**. **(B)** Effects of LPS and LETX-VI on Arg-1 level, detected by western blot analysis. Gray analysis was shown in **Supplementary Figure 3B**. **(C)** Effects of LPS and LETX-VI pretreatment on Arg-1 level, detected by laser confocal scanning microscopy. Relative fluorescence intensity was shown in **Supplementary Figure 3C**. n ≥ 3.

Macrophage mannose receptor (CD206) is one of the surface biomarkers of M2 macrophages (52) and we determined the effects of LPS and LETX-VI on its abundance level so as to probe into the ability of LETX-VI to promote RAW264.7 cell polarization towards M2 phenotype. The results (**Figure 4A**; **Supplementary Figure 3A**) indicated that, after treatment of the RAW264.7 cells with 1 µg/ml LPS for 12 h, the CD206 level was decreased compared with the control, whereas treatment with 4 µM LETX-VI alone for 12 h increased the level of CD206, suggesting that LPS leads to M1 activation and LETX-VI inhibits M1 RAW264.7 cells and promotes M2 polarization of the macrophages. Moreover, pretreatment of RAW264.7 cells with LETX-VI before LPS application could alleviate LPS-induced polarization of the macrophages towards M1 phenotype.

When the effects of LPS and LETX-VI on Arg-1, another biomarker of anti-inflammatory M2 macrophages, were determined using western blotting. the results (**Figure 4B**; **Supplementary Figure 3B**) showed that, compared with the control, treatment of RAW264.7 cells with 1 µg/ml LPS significantly reduced Arg-1 level, whereas 4 µM LETX-VI showed an opposite effect, again confirming the ability of LETX-VI to inhibit LPS-induced M1 macrophages and restore M2 macrophages. Similarly, pretreatment of the RAW264.7 cells with LETX-VI (0.1, 1 and 4 µM) attenuated LPS-caused decrease in Arg-1 level in a concentration-dependent manner, demonstrating that LETX-VI pretreatment could be used to inhibit M1 macrophage activation to a certain degree. In order to further verify the effects of LPS and LETX-VI on Arg-1 level in RAW264.7 cells, immunofluorescent staining of the Arg-1 was performed. The laser confocal scanning microscopy indicated that LPS led to a remarkable decrease in the fluorescence intensity for Arg-1, and pretreatment of the cells with LETX-VI (0.1, 1 and 4 µM) abolished the adverse influence of LPS on the fluorescence intensity for Arg-1, keeping the fluorescence intensity for Arg-1 to be comparable to that of the control (**Figure 4C**; **Supplementary Figure 3C**), supporting the conclusion on the effects of LPS and LETX-VI on Arg-1 level drawn with western blot analysis (**Figure 4B**; **Supplementary Figure 3B**).

### 3.5 LETX-VI protects PC12 cells by inhibiting LPS-induced inflammation

#### 3.5.1 LETX-VI attenuates the effects of LPS-induced inflammation on the viability, TH and Nurr1 of PC12 cells

**Figure 5:**
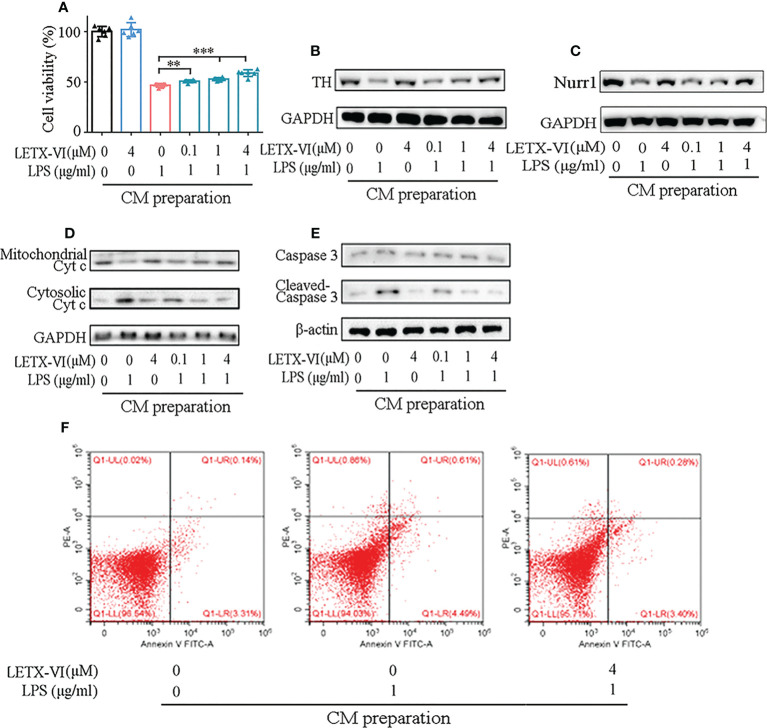
LETX-VI protects PC12 cells by inhibiting LPS-induced inflammation. **(A)** Effect of RAW264.7 cell CMs on PC12 cell viability. **(B)** Effect of RAW264.7 cell CMs on tyrosine hydroxylase (TH) level. Gray analysis was shown in **Supplementary Figure 4A**. **(C)** Effect of RAW264.7 cell CMs on Nurr1 level. Gray analysis was shown in **Supplementary Figure 4B**. **(D)** Effect of RAW264.7 cell CMs on the distribution of cytochrome c (Cyt c) between mitochondria and cytosol in PC12 cells. Gray analysis was shown in **Supplementary Figure 4C**. **(E)** Effect of RAW264.7 cell CMs on the levels of caspase 3 and activated caspase 3 (cleaved-casepase 3) in PC12 cells. Gray analysis was shown in **Supplementary Figure 4D**. **(F)** Representative dot plots of apoptotic rate analysis, showing the effect of RAW264.7 cell CMs on PC12 cell apoptosis. CM, conditioned medium. n ≥ 3. **P < 0.01, ***P < 0.001.

In order to further evaluate the efficiency of LETX-VI in protecting nerve cells through inhibiting inflammation, the effect of RAW264.7 cell conditioned medium (CM) prepared with or without LPS and LETX-VI treatment on PC12 cell viability were first determined. The results shown in the **Figure 5A** indicate that the RAW264.7 cell CM prepared with 4 μM LETX-VI treatment for 12 h had no significant effect on the viability of PC12 cells, whereas the CM prepared with 1 µg/ml LPS treatment for 12 h significantly reduced the viability of PC12 cells, suggesting that the LPS-induced inflammation heavily affected the viability of PC12 cells. When the PC12 cells were cultured with the CM prepared with LETX-VI (0.1, 1 and 4 µM) pretreatment for 12 h before LPS treatment, the viability of PC12 cells was enhanced as increasing the LETX-VI concentration compared with the cell viability without LETX-VI pretreatment (*P*< 0.01 and 0.001). These observations demonstrate that LETX-VI up to 4 µM itself has no obvious effect on the viability of PC12 cells and, however, LETX-VI pretreatment can provide a certain protective effect for the nerve cells in a concentration-dependent manner by alleviating LPS-evoked inflammation.

PC12 cells are a model for dopaminergic cells and can synthesize, store and release dopamine. Tyrosine hydroxylase (TH), as the key enzyme for the synthesis of dopamine, is closely related to the viability of the dopaminergic cells (53, 54). When we detected the effect of RAW264.7 cell CM on the TH level in PC12 cells, the CM prepared with 1 µg/ml LPS treatment was found to remarkably reduce TH level, and the CM prepared with LETX-VI pretreatment, like the situation in cell viability detection, mitigated LPS treatment CM-caused decrease in the TH level in a dose-dependent manner (**Figure 5B**; **Supplementary Figure 4A**).

In view of the report that nuclear receptor related protein 1 (Nurr1) may protect dopaminergic neurons from inflammation-induced death (9), we detected the effects of CMs prepared with LPS and LETX-VI treatment on Nurr1 level of PC12 cells. As shown in (**Figure 5C**; **Supplementary Figure 4B**), the CM prepared with 1 μg/ml LPS treatment obviously decreased the Nurr1 level, and that prepared with 4 µM LETX-VI alone had no obvious effect on the level of Nurr1. However, the CMs prepared by preteatment with LETX-VI (0.1, 1 and 4 µM) before LPS treatment had less adverse effects on Nurr1 level, and the 4 µM LETX-VI pretreatment-prepared CM led to the Nurr1 level to be comparable to that of the control. These results indicate that, although LETX-VI itself has no obvious effect on the Nurr1 level, LETX-VI pretreatment can effectively suppress the adverse effect of LPS-stimulated inflammation on Nurr1 level, which is helpful for Nurr1 to exert its neuroprotective effect.

#### 3.5.2 LETX-VI mitigates the induction effect of LPS-induced inflammation on apoptosis

To further reveal how LPS-induced inflammation of RAW264.7 cells affects PC12 cells and even better understand the neuroprotective effect of LETX-VI, we detected the effect of RAW264.7 cell CM on the apoptosis of PC12 cells. As shown in **Figure 5D** and **Supplementary Figure 4C**, compared with the control, culture of PC12 cells with RAW264.7 cell CM prepared with 1 µg/ml LPS treatment for 12 h increased the cytosolic Cyt c level of the PC12 cells, whereas 4 µM LETX-VI treatment-prepared CM had no obvious influence. When the CM prepared with LETX-VI (0.1, 1 and 4 µM) pretreatment for 12 h before LPS treatment was used to culture PC12 cells for 72 h, the cytosolic Cyt c level was decreased, with the mitochondrial Cyt c level being changed in an opposite tendency. **Figure 5E** and **Supplementary Figure 4D** shows that 1 µg/ml LPS treatment-prepared CM increased the level of cleaved caspase 3, the activated form of caspase 3, in PC12 cells, suggesting its promoting effect on apoptosis. 4 µM LETX-VI treatment-prepared CM did not obviously affect the levels of both caspase 3 and cleaved-caspase 3. Nevertheless, when the PC12 cells were cultured with the CMs prepared by pretreating RAW264.7 cells with LETX-VI at 0.1, 1 and 4 µM, respectively, the level of cleaved caspase 3 was gradually decreased to that of the control.

For further probing into the effect of CM on the apoptosis of PC12 cells, we used FITC-labeled annexin V and PI to treat the CM-cultured PC12 cells and directly analyzed the apoptotic rate with the flow cytometry. The results indicated that the apoptotic rates of PC12 cells cultured with the 1 µg/ml LPS treatment-prepared CM was significantly increased compared with the control (5.06 ± 0.76% vs 3.63 ± 1.65%) (*P*< 0.05). When the PC12 cells were cultured with the CM prepared by 4 µM LETX-VI pretreatment before LPS treatment, the apoptotic rate of PC12 cells was comparable to that of the control (3.88 ± 0.44 vs 3.63 ± 1.65%) (*P* > 0.05). **Figure 5F** shows the representative dot plots of apoptotic rate analysis.

All the observations demonstrate that LETX-VI may exert neuroprotective effects through attenuating or abolishing inflammation-caused caspase 3 activation, mitochondrial apoptosis and decrease in the expression of the proteins that have neuroprotective effects such as Nurr1.

### 3.6 LETX-VI attenuates LPS-induced depression in the depression mouse model

There have been a large number of reports revealing a close linkage between inflammation and depression (2, 4, 6, 55). Therefore, after LETX-VI was confirmed to be of potent anti-inflammation, its potential anti-depression effect was subsequently investigated with the depression mouse model.

#### 3.6.1 LETX-VI improves depressive behaviors of depression model mice

**Figure 6:**
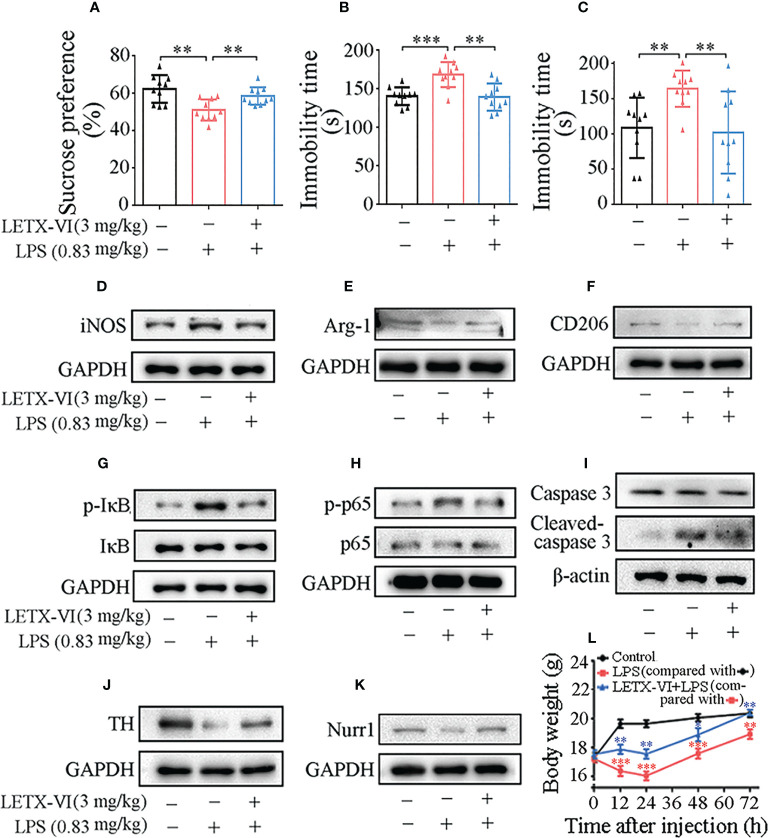
LETX-VI improves depressive behaviors and alleviates LPS-induced changes in the body weight and the levels of selected anti-inflammation and neuroprotection-related proteins in the brain of depression model mice. **(A–C)** Effects of intraperitoneal injection of LPS and pretreatment with LETX-VI before LPS injection on the sucrose preference and the immobility times in tail suspension and force swimming tests, respectively. **P< 0.01, ***P< 0.001. **(D–K)** Effects of intraperitoneal injection of LPS and pretreatment with LETX-VI before LPS injection on the levels of selected anti-inflammation and neuroprotection-related proteins in the brain of depression model mice. iNOS, inducible nitric oxide synthase. Arg-1, arginase-1. CD206, macrophage mannose receptor. p-IκB, phosphorylated IκB. IκB, inhibitory protein/nuclear transcription factor-κB (NF-κB) complex. p-P65, phosphorylated p65. p65, one protein in p65-p50 heterodimer (inactive NF-κB). TH, tyrosine hydroxylase. Nurr1, nuclear receptor related protein 1. Gray analysis was shown in **Supplementary Figures 5A–H**. **(L)** Effects of LPS and LETX-VI on the body weight of mice. n ≥ 3. **P< 0.01, ***P< 0.001.

After the mice were adapted to the experimental environment, the amounts of sucrose solution and water consumed during a 24 h period were recorded for evaluating the sucrose preference of the tested mice. As a result, compared with the control intraperitoneally injected with physiological saline, intraperitoneal injection of LPS at a dose of 0.83mg/kg body weight significantly reduced the sucrose preference of the mice (*P*< 0.01) and, however, pretreatment with LETX-VI (3 mg/kg body weight) prior to LPS injection abolished the LPS-induced decrease in sucrose preference (*P*< 0.01), making the sucrose preference of the mice comparable to that of the control (**Figure 6A**). During the tail suspension test, the LPS-treated mice exhibited significantly longer time immobile than the control (*P*<0.001). Pretreatment with LETX-VI remarkably alleviated the LPS-stimulated increase in the immobility period (*P<* 0.01), making the time immobile of the mice comparable to that of the control (**Figure 6B**). Similar observations were obtained in the forced swimming test. As shown in **Figure 6C**, LPS-treated mice showed significant longer immobility time in water than the control mice (*P*<0.01). Pretreatment with LETX-VI inhibited the LPS-caused increase in immobility period and led to the immobility period being comparable to that of the control mice. All the results indicate that LETX-VI pretreatment mitigates the depression behaviors of LPS-induced depression model mice and suggest the anti-depression effect of LETX-VI.

#### 3.6.2 Western blot analysis of the effects of LETX-VI on proteins in mouse brain tissues

Besides detection of the effect of LETX-VI on the depression behaviors of model mice, the effects of LETX-VI pretreatment on the representative proteins that are related with anti-inflammation and neuroprotection in the mouse brain tissues were also investigated, so as to compare *in vivo* and *in vitro* experimental results. The results shown in **Figure 6D** and **Supplementary Figure 5A** indicate that intraperitoneal injection of LPS (0.83 mg/kg body weight) resulted in a significant increase in the level of iNOS, used as a representative pro-inflammatory factor in this assay; pretreatment with LETX-VI (3 mg/kg body weight) twice before LPS injection eliminated such an increase in iNOS level, demonstrating that LETX-VI could exert anti-inflammation effect under both *in vitro* and *in vivo* experimental conditions. The levels of Arg-1 and CD206 protein, representative anti-inflammatory factor and biomarker of M2 activation macrophages/microglia (56), in mouse brain tissues were reduced by LPS, and LETX-VI preteatment alleviated the adverse effect of LPS (**Figures 6E, F**; **Supplementary Figures 5B, C**). These results confirm that LETX-VI, like the situation *in vitro* experiments, has the ability to resist LPS-evoked inflammatory and facilitate the shift of microglial M1 to M2 phenotype.

**Figures 6G, H** and **Supplementary Figures 5D, E** show that the levels of p-IκB and p-p65 were remarkably increased by LPS treatment and LETX-VI pretreatment attenuated the increase amplitude. In order to detect the *in vivo* neuroprotective effect of LETX-VI, the levels of caspase 3, cleaved caspase 3 and TH in the mouse brain tissues were measured. The results showed that both LPS and LETX-VI have no obvious effects on the total level of caspase 3. However, LPS increased the cleaved casepase 3 level, whereas LETX-VI pretreatment mitigated the increase amplitude of cleaved caspasse 3 (**Figure 6I**; **Supplementary Figure 5F**). TH level was remarkably decreased by LPS and however LETX-VI pretreatment efficiently alleviated the decrease amplitude (**Figure 6J** and **Supplementary Figure 5G**). **Figure 6K**; **Supplementary Figure 5H** shows that LPS decreased the Nurr1 level in mouse brain tissues; however, LETX-VI pretreatment eliminated such an adverse effect of LPS on Nurr1.

All the results demonstrate that LETX-VI pretreatment, like that in the *in vitro* experiments, attenuates or abolishes LPS-induced alternations in the levels of the proteins related with inflammation and neuroprotection.

#### 3.6.3 Effects of LPS and LETX-VI on the body weight of mice

When the changes in the mouse body weight during 72 h after injection of drugs or physiological saline were measured, it was found that the body weight of the mice in control group was rapidly increased during the first 12-h period after physiological saline injection, followed by a slight rising till 72 h (**Figure 6L**). Compared with the control, intraperitoneal injection of LPS (0.83 mg/kg body weight) resulted in a significant decrease in body weight particularly in the first 24-h period (P< 0.001 or 0.01). Pretreatment of the mice with LETX-VI (3 mg/kg body weight) twice prior to LPS injection significantly attenuated the LPS-caused body weight decrease and led to the body weight of the mice to be comparable to that of the control mice at 72 h (**Figure 6L**). These observations suggest that LPS may decrease the body weight of the mice by inducing depression to decrease food intake and interfering with the metabolism of substance and energy; pretreatment with LETX-VI may markedly mitigate such adverse effects caused by LPS.

#### 3.6.4 Immunofluorescent and Nissl staining analyses of LETX-VI abating the adverse effects of LPS on mouse brain cells and proteins

**Figure 7:**
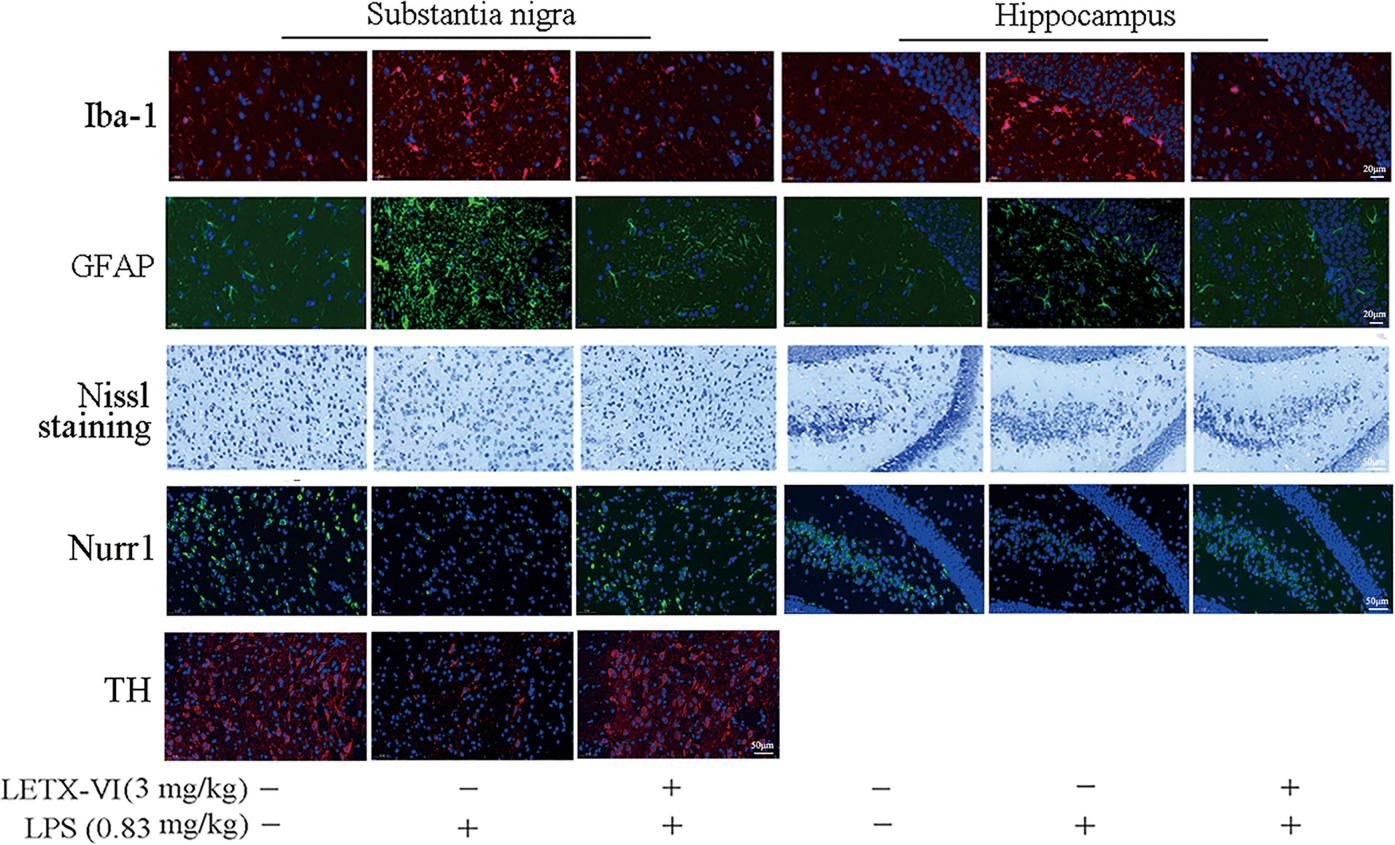
Immunofluorescent and Nissl staining analyses of LETX-VI abating the adverse effects of LPS on mouse brain cells and proteins. Iba-1, ionized calcium binding adaptor molecule-1. GFAP, glial fibrillary acidic protein. Nurr1, nuclear receptor related protein 1. TH, tyrosine hydroxylase. Relative fluorescence intensity of Iba-1 was shown in **Supplementary Figure 6A**. Relative fluorescence intensity of GFAP was shown in **Supplementary Figure 6B**. Number of Nissl-positive neurons/area was shown in **Supplementary Figure 6C**. Relative fluorescence Intensity of Nurr1 was shown in **Supplementary Figure D**. Relative fluorescence intensity of TH was shown in **Supplementary Figures 6A, E**. n ≥ 3.

For detecting the *in vivo* effects of LPS and LETX-VI on mouse brain inflammation, the levels of ionized calcium binding adaptor molecule-1(Iba-1) and glial fibrillary acidic protein (GFAP), biomarkers for the activation of microglia and astrocytes, respectively (57), in the substantia nigra and hippocampus were determined with immunofluorescent staining. Laser confocal scanning microscopy results (**Figure 7**; **Supplementary Figures 6A, B**) showed that intraperitoneal injection of LPS (0.83 mg/kg body weight) resulted in increased intracellular fluorescence intensity for Iba-1 and GFAP in both substantia nigra and hippocampus, suggesting that LPS induced the activation of microglia and astrocytes. Injection of LETX-VI (3 mg/kg body weight) before LPS injection attenuated or eliminated LPS-induced increase in the fluorescence intensity for the two biomarker proteins, demonstrating that LETX-VI pretreatment suppressed LPS-evoked activation of the microglia and astrocytes. These results indicate that LETX-VI pretreatment may inhibit LPS-induced inflammation and thus provide a certain protective effect for the neurons.

To further confirm the protective effect of LETX-VI on neurons, Nissl staining was employed to monitor the changes in the number of alive neurons in the substantia nigra and hippocampus. As shown in **Figure 7** and **Supplementary Figure 6C**, LPS treatment resulted in a decrease in the number of alive neurons in both substantia nigra and hippocampus, and LETX-VI pretreatment mitigated the adverse influence caused by LPS. Nurr1 is closely related with anti-inflammation and neuroprotection and reduced Nurr1 level resulted in exaggerated inflammatory responses in microglia, which were further amplified by astrocytes, and led to the production of factors that caused death of dopaminergic neurons (9). When we detected the effects of LPS and LETX-VI pretreatment on Nurr1 level with immunofluorescent staining, the intracellular fluorescence intensity for Nurr1 in both substantia nigra and hippocampus was markedly lowered by LPS, and LETX-VI pretreatment mitigated the influence of LPS, making the fluorescence intensity for Nurr1 comparable to that of the control (**Figure 7**; **Supplementary Figure 6D**). These results suggest that LETX-VI pretreatment may protect dopaminergic neurons through inhibiting LPS-induced decrease in Nurr1 level. Immunofluorescent staining analysis indicated that LPS treatment led to a significant decrease in the intracellular fluorescence intensity for TH in substantia nigra and pretreatment with LETX-VI before LPS injection made the fluorescence intensity comparable to that of the control (**Figure 7**; **Supplementary Figure 6E**), confirming the protective effect of LETX-VI pretreatment on dopaminergic neurons, which is in consistence with the conclusion drawn with western blot analysis (**Figure 6J**; **Supplementary Figure 5G**).

### 3.7 Safety assessment of the application of LETX-VI to mice

**Figure 8:**
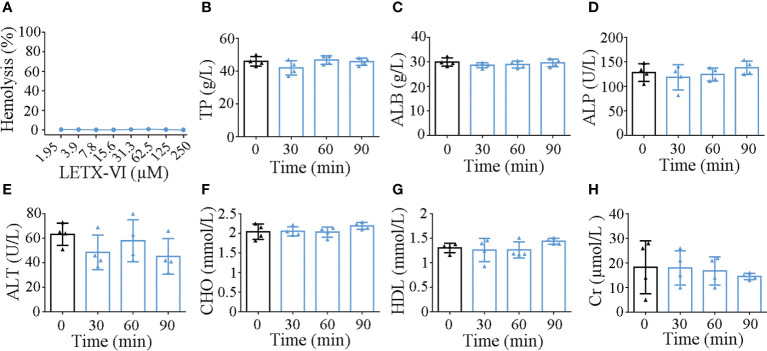
Effects of LETX-VI on red blood cells **(A)** and some blood biochemical indexes **(B–H)** of mice. TP, serum total protein; ALB, albumin; ALP, alkaline phosphatase; ALT, glutamate pyruvate transaminase; CHO, cholesterol; HDL, high-density lipoprotein; Cr, creatinine. n ≥ 3.

When the hemolytic activity of LETX-VI toward mouse red blood cells was determined, LETX-VI at concentrations of up to 250 µM was found to have no obvious hemolytic activity under the present experimental conditions (**Figure 8A**). At the different time points (30, 60 and 90 min) after the injection of LETX-VI at a dose of 3 mg/kg body weight, the levels of serum total protein, albumin, alkaline phosphatase, glutamate pyruvate transaminase, cholesterol, high-density lipoprotein and creatinine were quantitatively determined. The results showed that, compared with the control, up to 90 min after LETX-VI treatment the levels of those blood biochemical indexes were not significantly altered, although there were a certain degree of fluctuations (**Figures 8B–H**). In view of the fact that most of the detected blood indexes are closely related with the physiological functions of liver, kidney, biliary system, etc., and these indexes are not obviously influenced by LETX-VI, suggesting that application of LETX-VI at 3 mg/kg body weight under our present experimental conditions is not toxic toward the mice and hint the safety of LETX-VI application in the future.

## 4 Discussion

Macrophages are important immune cells, which participate in the modulation of a series of inflammations and autoimmune diseases through either specific (cellular immunity) or non-specific defenses (innate immunity) *in vivo* (58). Inflammatory responses induced by LPS in macrophages have been commonly used as a classical model to evaluate the anti-inflammatory activity of active substances (20–22, 59). Macrophages can be polarized into classically activated macrophages (M1) and alternatively activated macrophages (M2) in the immune system, performing pro-inflammatory and anti-inflammatory functions, respectively (59). When we used RAW263.7 macrophages as an *in vitro* cell model to investigate the anti-inflammatory effect of LETX-VI, we found that, although LETX-VI itself has no obvious influence on the RAW264.7 cells, pretreatment of the macrophages with LETX-VI mitigates or reverses LPS-induced increase in pro-inflammatory factors including NO, iNOS, TNF-α and IL-6, and at the same time alleviates the inhibitory effects of LPS on CD206 and Arg-1. These results suggest that LETX-VI has a potential to reduce over-production of free radicals and inhibit excessive inflammation, and thus shows promising anti-inflammatory and neuroprotective effects, because dysregulated neuroinflammation can result in altered metabolism, increased demyelination, neuronal apoptosis, neuronal autophagy and perturbed mitochondrial energetics that compromise the functioning of nerve cells and ultimately cause their death (60, 61). Besides, the inflammation induced experimentally in rodents can reduce rates of neurogenesis, cause dendritic atrophy of pyramidal neurons and alter density and stability of neuronal spines and synapses (62, 63). In our present study, to confirm the neuroprotective effect of LETX-VI, we employed RAW264.7 cell conditioned medium to culture PC12 cells, a commonly used neuron model. The results demonstrated that the conditioned medium prepared with LPS treatment displayed obvious adverse influence on the PC12 cells, and the conditioned medium prepared with LETX-VI pretreatment before LPS application showed much weaker influence on the PC12 cells. These results have experimentally demonstrated that LETX-VI pretreatment provides certain protection for neurons by inhibiting the excessive inflammation.

NF-κB (p65-p50 heterodimer) is known to up-regulate the expressions of cytokines, chemokines, inducible effectors enzymes, etc, typically involved in immunity and inflammation. In resting cells, the p65-p50 dimer is complexed with its inhibitor IκB, and retained in the cytosol. IκB can be phosphorylated by IKK, a kinase for IκB. Phosphorylation of IκB is followed by ubiquitination and degradation by proteasomes, releasing the p65-p50 heterodimer from the inhibitory complex (64–66). The free heterodimer enters the nucleus to bind to the specific sites on DNA via p65 and promotes the expression of multiple inflammatory factors (50). P65 can also be phosphorylized in both cytosol and nucleus in response to a variety of stimuli (64, 67). Elevated level of phosphorylated P65 (p-p65) in the nucleus is favorable for p65 binding to transcriptional coactivators and promoting transcription (50). Thus, activation of NF-κB signaling pathway is a key event in pro-inflammatory signal transduction, and sustained hyper-activation of NF-κB dysregulates immune signaling and inflammatory reactions (7). In addition, there are other proteins synergizing with NF-κB to regulate the expression of inflammatory factors. For example, Nurr1 exerts anti-inflammatory effects by docking to NF-κB-p65 on target promoters of inflammatory genes such as those for TNF-α and iNOS, and recruits the CoREST corepressor complex to result in clearance of NF-κB-p65 and transcriptional repression (9). Therefore, inhibition of the activation of NF-κB signaling pathway and the expression of related proteins can suppress expression of the genes for multiple inflammatory factors and mitigate their adverse influence on neurons.

Our experimental results showed that LPS activates NF-κB signaling pathway by promoting the phosphorylation of IκB and p65, which is in consistence with the relevant reports (40, 68). LETX-VI pretreatment inhibits LPS-induced NF-κB signaling pathway activation by suppressing the phosphorylation of IκB and p65 and thus inhibits LPS-induced inflammation. Besides, LETX-VI abolishes the inhibitory effect of LPS on Nurr1 level, which is favorable for Nurr1 to exert anti-inflammatory effects (9). These observations demonstrate that the anti-inflammatory and neuroprotective effects of LETX-VI involve the inhibition of NF-κB signaling pathway activation and the regulation of other inflammation-related protein expression. In addition, it is worthy of mentioning that we firstly detected the potential cytotoxicity of LPS and LETX-VI, and the results indicated that the cytotoxicity of LPS and LETX-VI at the concentrations used in the present study toward the RAW264.7 cells was limited, and their influences on the cells were primarily based on their effects on the metabolic and regulatory processes.

Numerous reports have shown that administration of LPS in rats or mice leads to sickness behaviors characterized by symptoms including lethargy, decreased food intake, locomotor activity, anhedonia, sleep disturbances, and increased sensitivity to pain. Some of these symptoms are thought to be very similar to clinically relevant symptoms of depression in humans (17, 69, 70). Therefore, systemic administration of LPS is frequently used to study inflammation-associated depression in rodents. Acute activation of the peripheral or central innate immune system in laboratory animals through the administration of LPS induces depressive-like behaviors, as usually measured by forced swim test, tail suspension test and sucrose preference test (25, 40, 71). When we utilized the LPS-induced mouse model of depression to investigate the *in vivo* anti-inflammation and anti-depression effects of LETX-VI, it was found that intraperitoneal injection of LETX-VI before LPS administration effectively alleviated LPS-induced decrease in sucrose preference and reversed LPS-caused increase in the immobility periods during the tail suspension and forced swimming tests, indicating the improving effect of LETX-VI on the depressive behaviors.

Considering the fact that acute systemic LPS administration induces pro-inflammatory cytokine release and neuroinflammation in the central nervous system (72, 73), we also detected the related changes in brain tissues of depression model mice treated with LPS and LETX-VI. The results demonstrated that LETX-VI pretreatment, like the situation in the *in vitro* experiments with macrophages, countered LPS-induced changes in levels of the proteins related with inflammation and neuroprotection. Immunofluorescent and Nissl staining analyses further confirmed such counteractions of LETX-VI. Particularly, immunofluorescent staining analysis demonstrated that LETX-VI pretreatment prevented TH and dopaminergic neurons from the adverse influence of LPS, suggesting that LETX-VI may promote the synthesis and release of dopamine that is implicated in depression and other dopamine-related diseases (33, 34). These results are consistent with our previous reports that LETX-VI is able to enter PC12 cells to promote the synthesis and release of dopamine (30, 31).

Taken together, our data demonstrate that application of LETX-VI alone at the experimental concentrations has been shown not to obviously influence RAW264.7 cells. However, pretreatment of the macrophages with LETX-VI before LPS application promotes M1-to-M2 phenotype shift, suppresses LPS-induced increase of pro-inflammatory factors, including IL-6, TNF-α, NO and iNOS, and alleviates the inhibitory effect of LPS on the expression of antiinflammation-related proteins such as Arg-1 and Nurr1. The anti-inflammation mechanism of LETX-VI involvs its inhibiting LPS-evoked NF-κB signaling pathway activation. Owing to its potent anti-inflammatory effects, LETX-VI is able to protect nerve cells and prevent inflammation-related psychiatric disorders including depression. Both *in vitro* and *in vivo* experiment results consistently demonstrate the anti-inflammation and neuroprotection effects of LETX-VI, which not only deepens our understanding of the mechanism and action of LETX-VI, but also provides new clues for the further researches including those on its potential applications in the fields of neurology and medicine.

## Data availability statement

The original contributions presented in the study are included in the article/**Supplementary Material**. Further inquiries can be directed to the corresponding author.

## Ethics statement

The study was performed in accordance with the recommendations of the Guide for the Care and Use of Laboratory Animals of the China National Institute of Health and the Ethics Committees of Hunan Normal University ratified all the experiments with animals.

## Authors contributions

Conceptualization: XW and HW. Designed and performed main experiments, prepared figures and wrote manuscript draft: HW and XW. Participated in designing and performing partial experiments, preparing samples and collecting data: YZ, ZL, SC, MS, PY, and ZD. Funding acquisition: XW. Project administration and supervision: XW. Manuscript draft review and editing: XW. All authors contributed to the article and approved the submitted version.
