# Supplementary material for: Latroeggtoxin-VI protects nerve cells and prevents depression by inhibiting NF-κB signaling pathway activation and excessive inflammation
Source: Front Immunol. 2023 May 15;14:1171351. doi: 10.3389/fimmu.2023.1171351 (PMC10225626; doi:10.3389/fimmu.2023.1171351)
Supplement: Supplementary file 1 [file DataSheet_1.pdf]

Supplementary Figs. Analysis of gray and relative fluorescence intensity

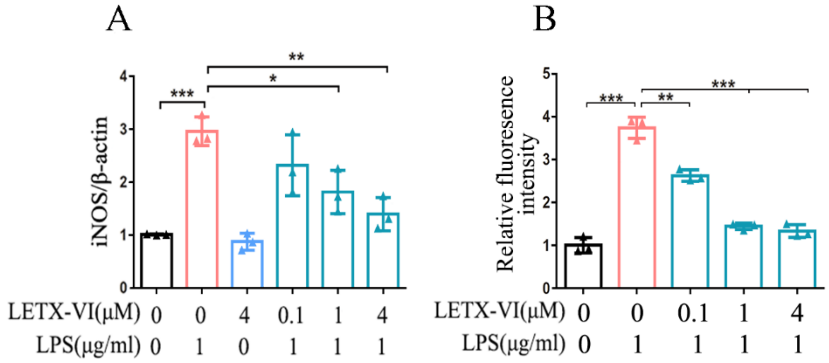

Supplementary Figure 1. Analysis of gray and relative fluorescence intensity of Figure 2.

*P < 0.05, ** P < 0.01, ***P < 0.001. n ≥ 3.

(**A**) Gray analysis of Figure 2C. Effects of LPS and LETX-VI on iNOS level.

(**B**) Relative fluorescence intensity of Figure 2D. Analysis of the effects of LPS and LETX-VI on iNOS level by laser confocal scanning microscopy.

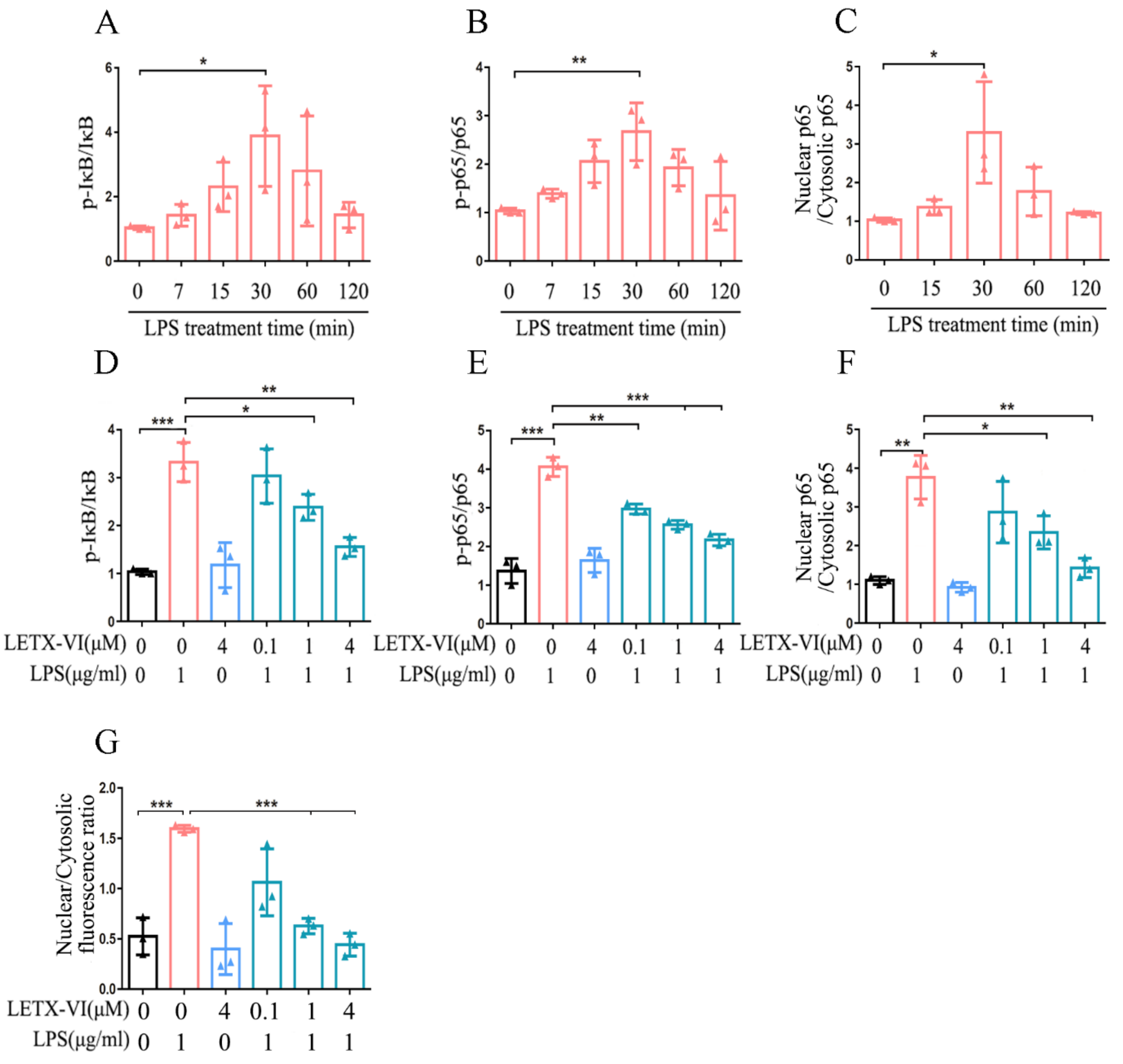

Supplementary Figure 2. Analysis of gray and relative fluorescence intensity of Figure 3.

*P < 0.05, ** P < 0.01, ***P < 0.001. n ≥ 3.

(**A**) Gray analysis of p-IκB in Figure 3A. Reatment of RAW264.7 cells with 1 μg/ml LPS for 30 min or so promoted the phosphorylation of IκB, leading to increased phosphorylated IκB (p-IκB).

(**B**) Gray analysis of p-p65 in Figure 3A. Reatment of RAW264.7 cells with 1 μg/ml LPS for 30 min or so promoted the phosphorylation of S536 in p65, leading to increased S536 in p65 (p-p65).

(**C**) Gray analysis of Figure 3B. LPS treatment promoted the entry of p65 into the nucleus

from cytosol of RAW264.7 cells.

(**D**) Gray analysis of IκB in Figure 3C. LETX-VI pretreatment inhibited the phosphorylation of IκB induced by LPS treatment for 30 min.

(**E**) Gray analysis of p-p65 in Figure 3C. LETX-VI pretreatment inhibited the S536 in p65 induced by LPS treatment for 30 min.

(**F**) Gray analysis of Figure 3D. LETX-VI pretreatment inhibited the entry of p65 into nucleus induced by LPS treatment for 30 min.

(**G**) Nuclear/Cytosolic flurescence ratio of Figure 3E. Immunofluorescent staining further confirmed that LETX-VI pretreatment inhibited the entry of p65 into nucleus induced by LPS treatment for 30 min.

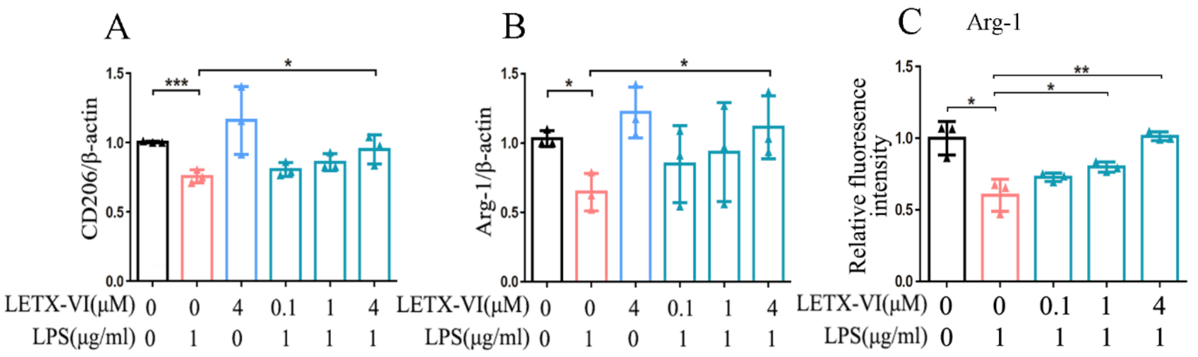

Supplementary Figure 3. Analysis of gray and relative fluorescence intensity of Figure 4.

*P < 0.05, ** P < 0.01, ***P < 0.001. n ≥ 3.

(**A**) Gray analysis of Figure 4A. Effects of LPS and LETX-VI on CD206 level, detected by western blot analysis.

(**B**) Gray analysis of Figure 4B. Effects of LPS and LETX-VI on Arg-1 level, detected by western blot analysis.

(**C**) Relative fluorescence intensity of Figure 4C. Effects of LPS and LETX-VI pretreatment on Arg-1 level, detected by laser confocal scanning microscopy.

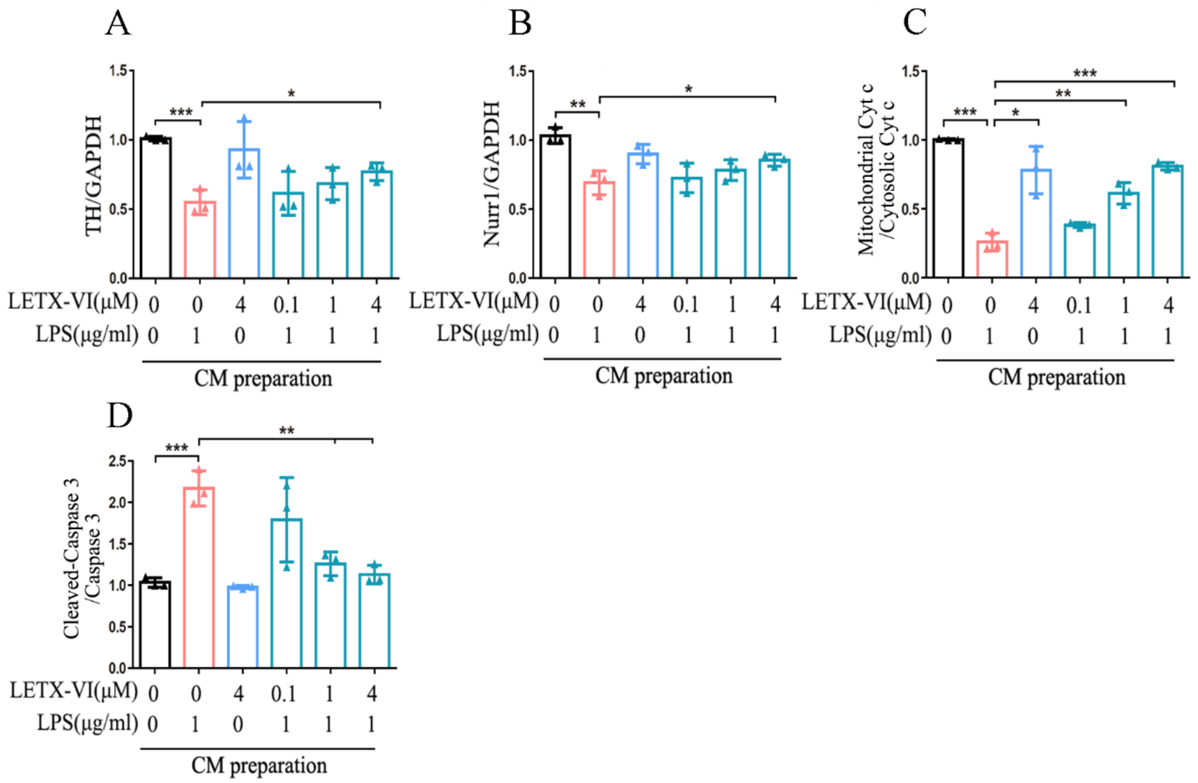

Supplementary Figure 4. Gray analysis of Figure 5. *P < 0.05, ** P < 0.01, ***P < 0.001. n ≥ 3.

(**A**) Gray analysis of Figure 5B. Effect of RAW264.7 cell CMs on tyrosine hydroxylase (TH) level.

(**B**) Gray analysis of Figure 5C. Effect of RAW264.7 cell CMs on Nurr1 level.

(**C**) Gray analysis of Figure 5D. Effect of RAW264.7 cell CMs on the distribution of cytochrome c (Cyt c) between mitochondria and cytosol in PC12 cells.

(**D**) Gray analysis of Figure 5E. Effect of RAW264.7 cell CMs on the levels of caspase 3 and activated caspase 3 (cleaved-casepase 3) in PC12 cells.

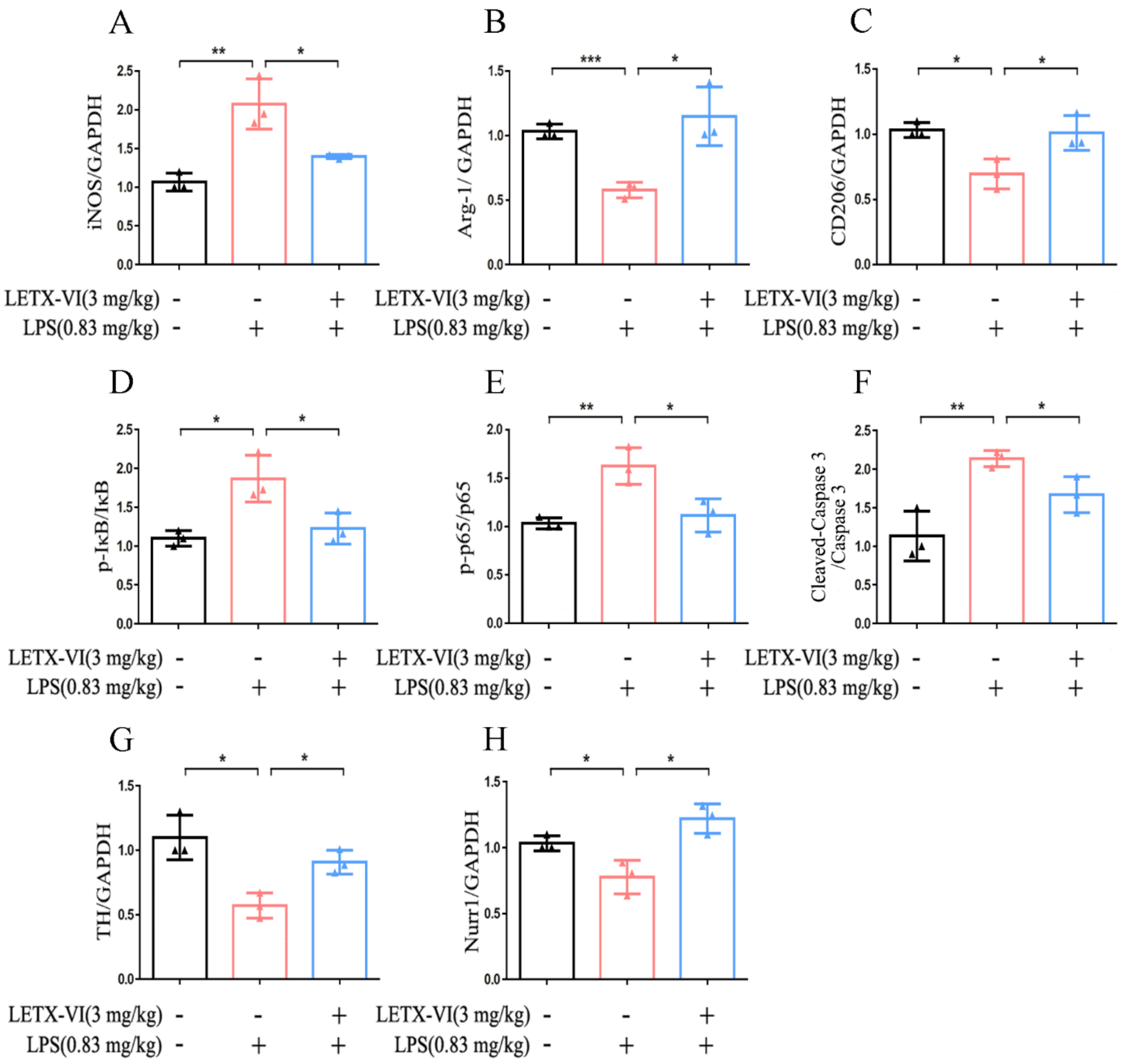

Supplementary Figure 5. Gray analysis of Fig. 6. *P < 0.05, ** P < 0.01, ***P < 0.001. n ≥ 3.

Figures. 6D-6K: Effects of intraperitoneal injection of LPS and pretreatment with LETX-VI before LPS injection on the levels of selected anti-inflammation and neuroprotection-related proteins in the brain of depression model mice.

(**A**) Gray analysis of Figure 6D. (**B**) Gray analysis of Figure 6E.

(**C**) Gray analysis of Figure 6F. (**D**) Gray analysis of Figure 6G.

(**E**) Gray analysis of Figure 6H. (**F**) Gray analysis of Figure 6I.

(**G**) Gray analysis of Figure 6J. (**H**) Gray analysis of Figure 6K.

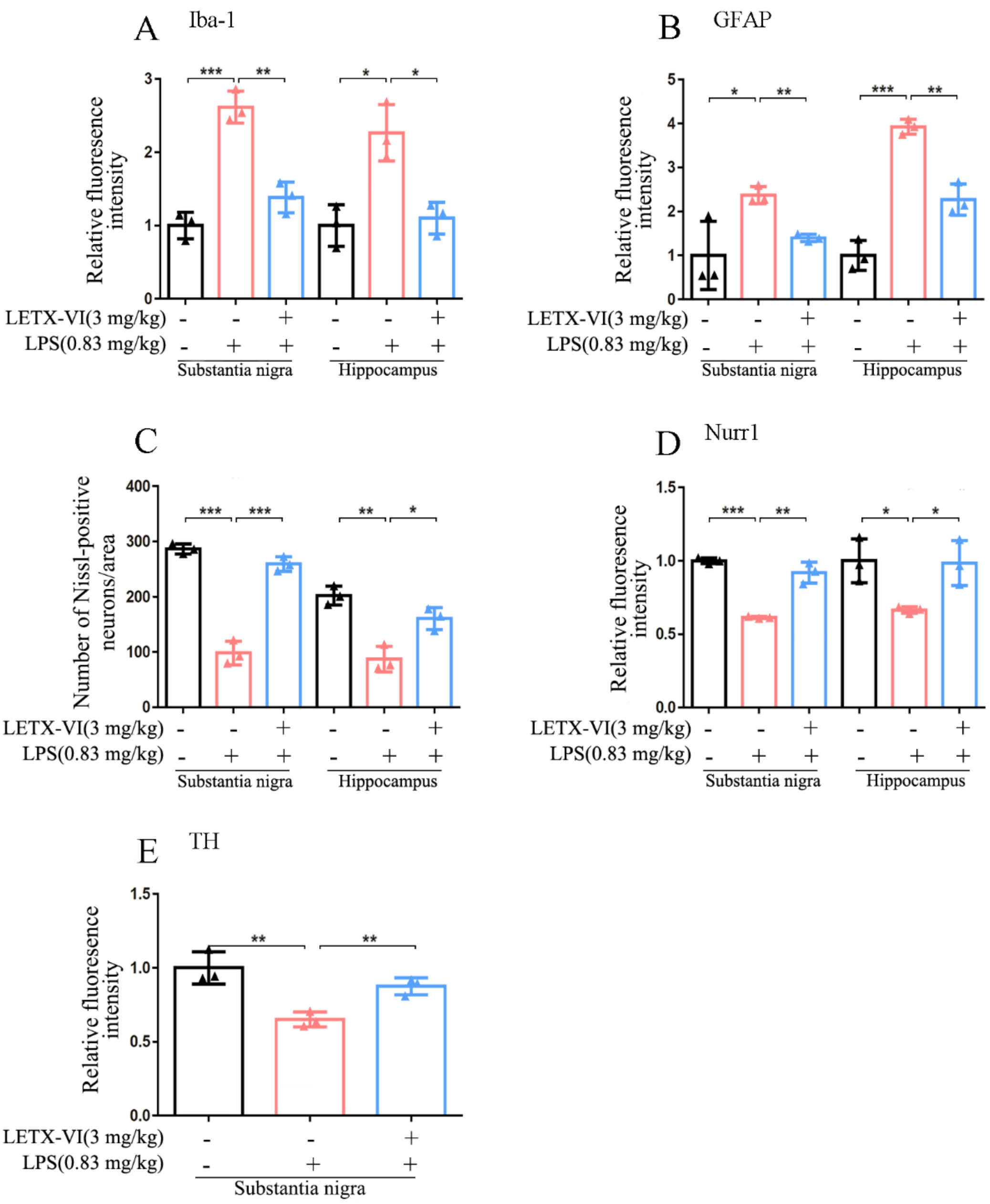

Supplementary Figure 6. Relative fluorescence intensity of Figure 7. *P < 0.05, ** P < 0.01, ***P < 0.001. n ≥ 3.

(**A**) Relative fluorescence intensity of Iba-1 in Figure 7.

(**B**) Relative fluorescence intensity of GFAP in Figure 7.

(**C**) Number of Nissl-positive neurons/area of Figure 7.

(**D**) Relative fluorescence intensity of Nurr1 in Figure 7.

(**E**) Relative fluorescence intensity of TH in Figure 7.
