# Supplementary material for: Latroeggtoxin-VI protects nerve cells and prevents depression by inhibiting NF-κB signaling pathway activation and excessive inflammation
Source: Front Immunol. 2023 May 15;14:1171351. doi: 10.3389/fimmu.2023.1171351 (PMC10225626; doi:10.3389/fimmu.2023.1171351)
Supplement: Supplementary file 2 [file DataSheet_2.pdf]

# Supplementary Figures. Raw images

Fig. 2c

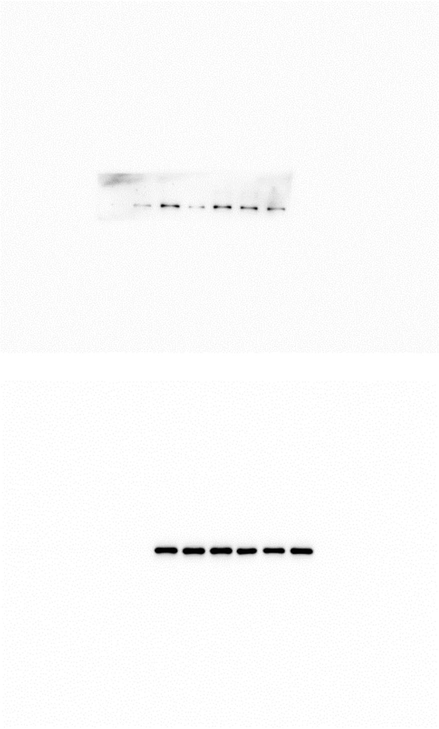

Fig. 3a

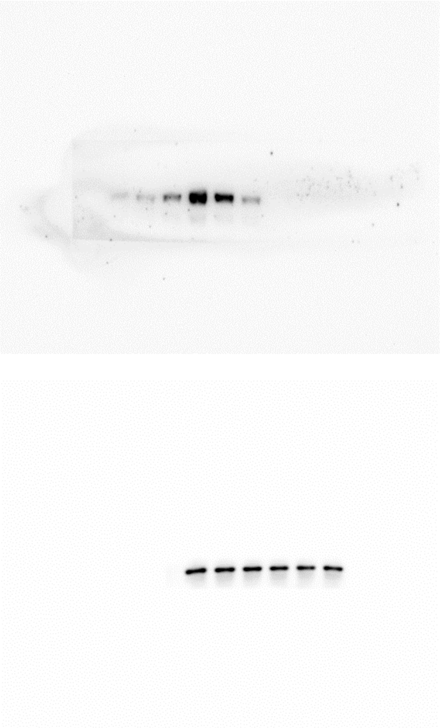

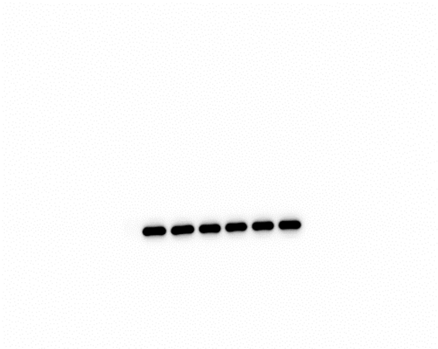
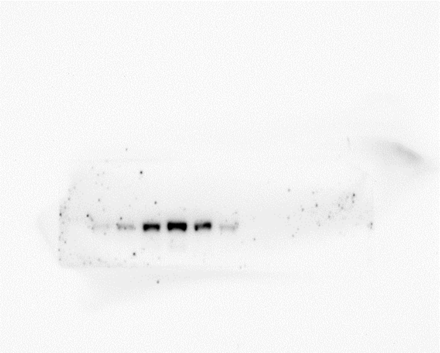
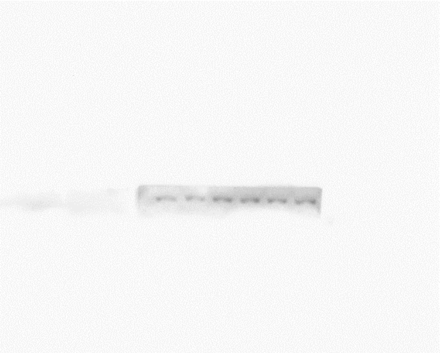
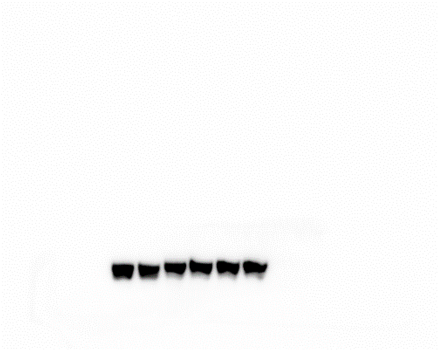

Fig. 3b

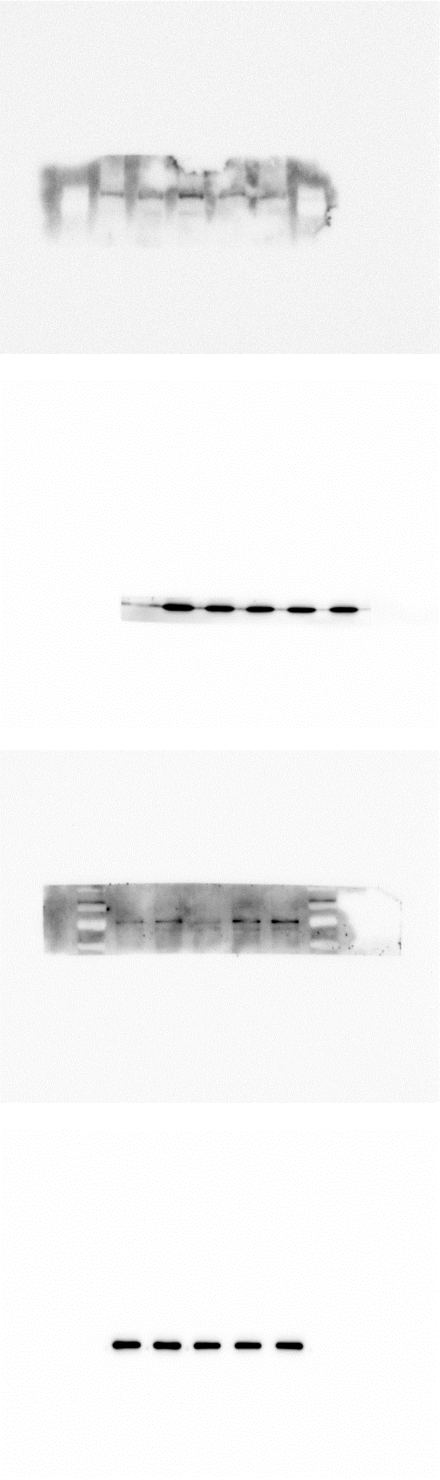

Fig. 3c

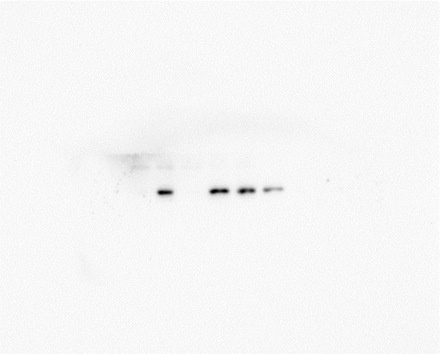

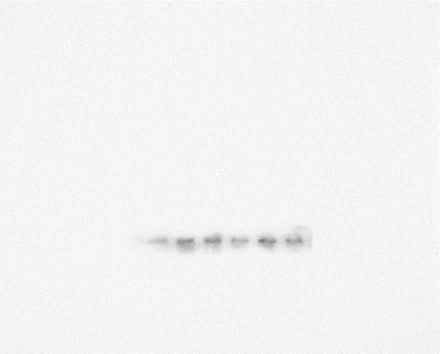



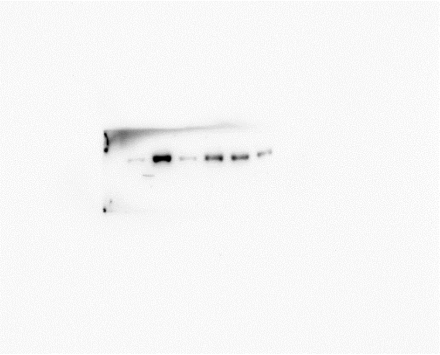

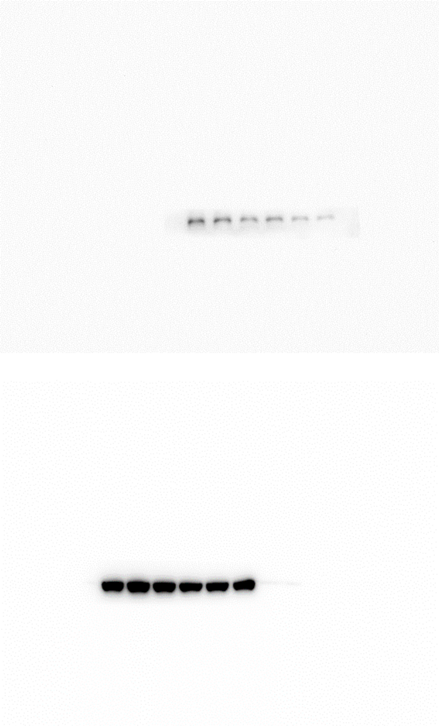

Fig. 3d

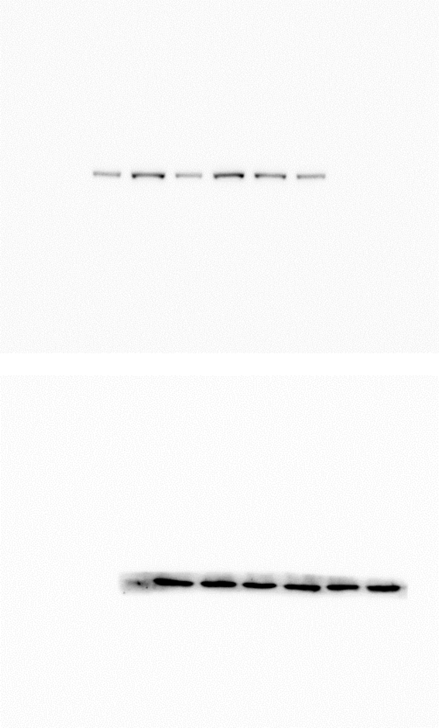

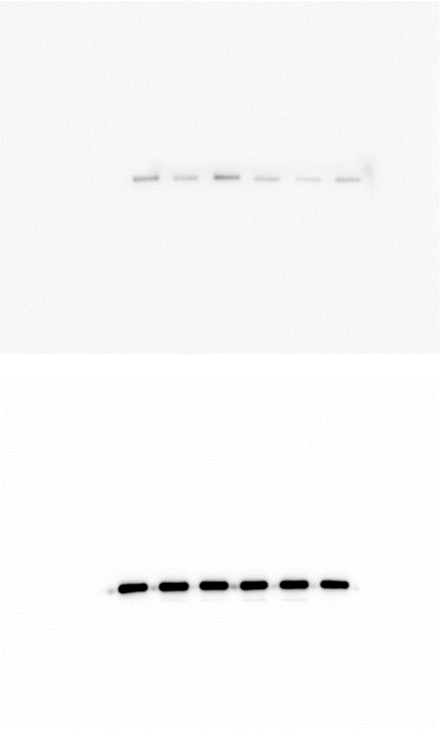

Fig. 4a

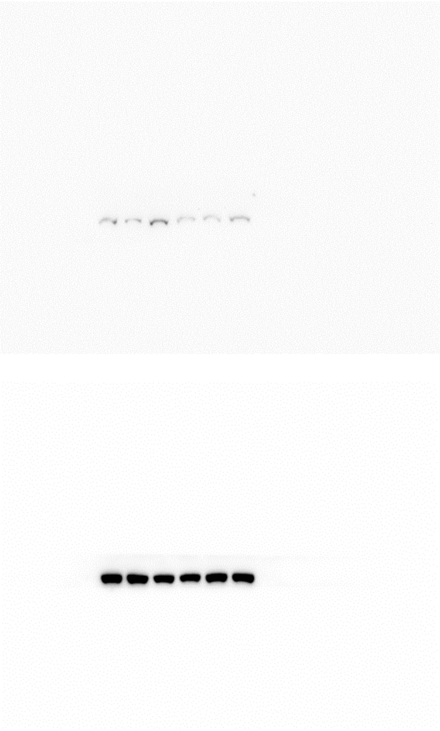

Fig. 4b

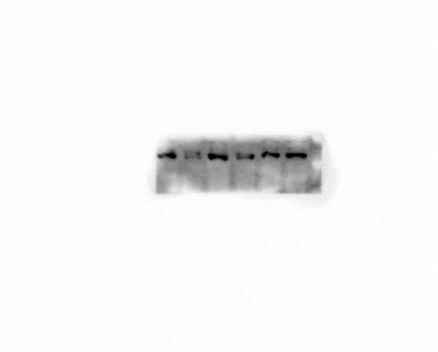



Fig. 5b



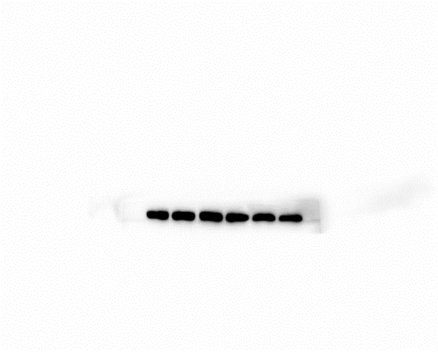

Fig. 5c

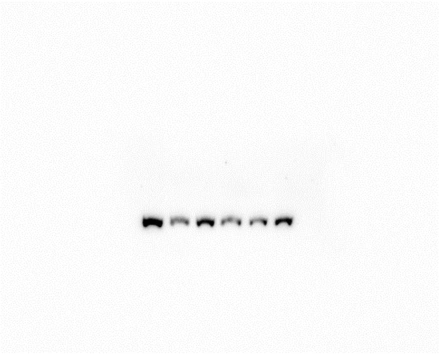

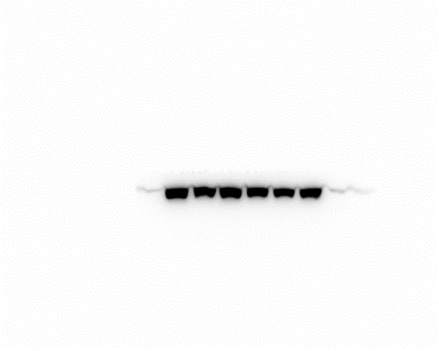

Fig. 5d

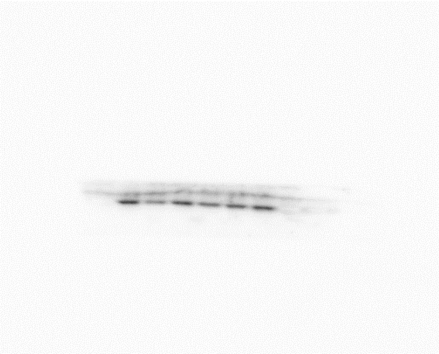

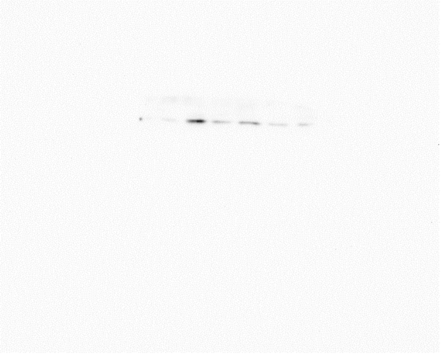

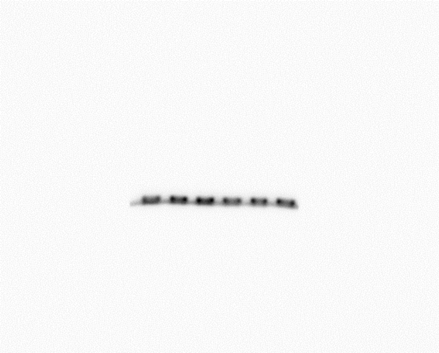

Fig. 5e

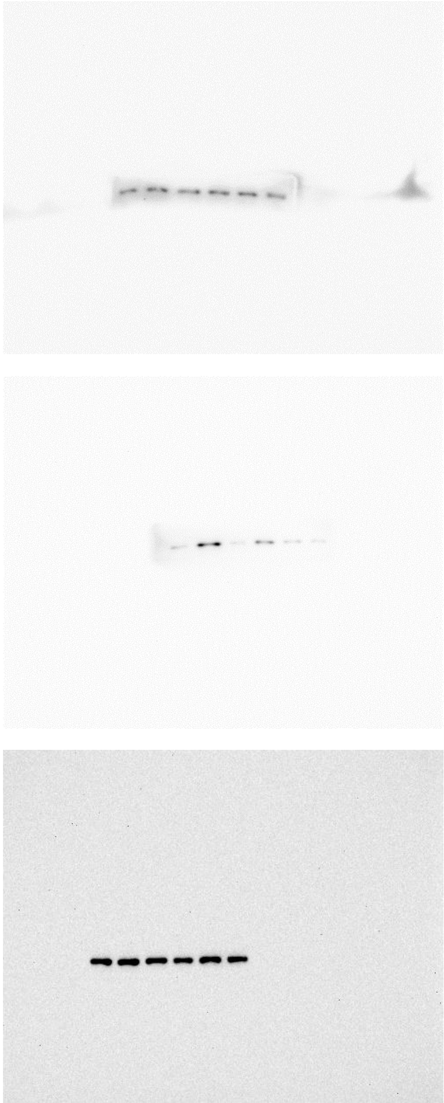

Fig. 6d

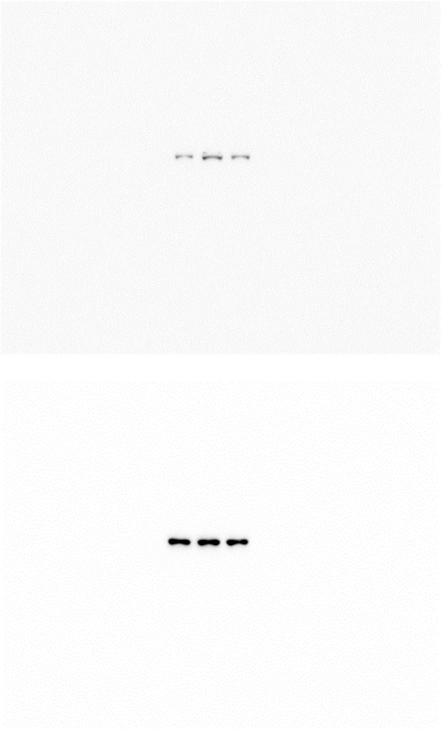

Fig. 6e

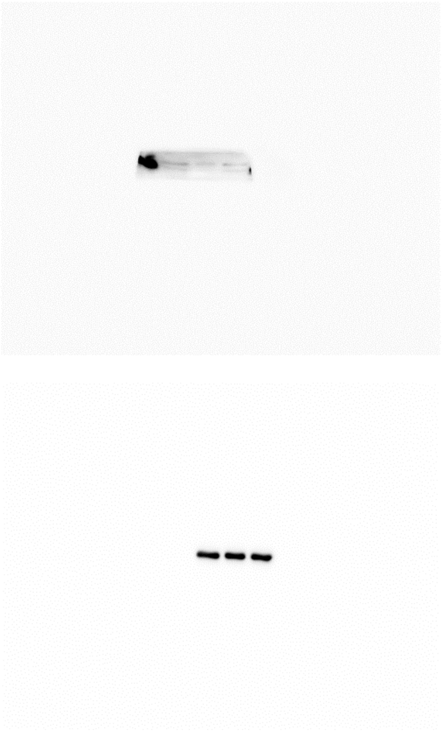

Fig. 6f

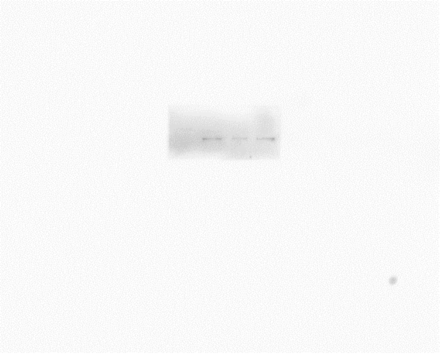



Fig. 6g

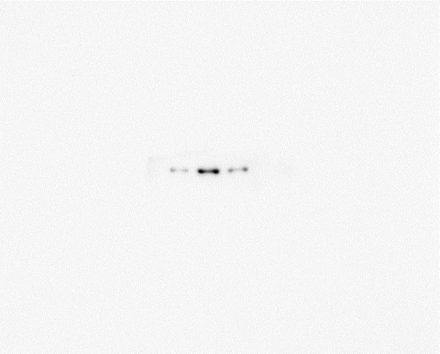

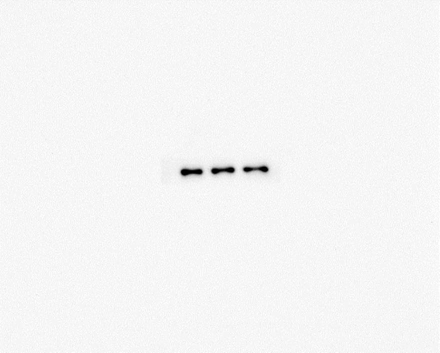

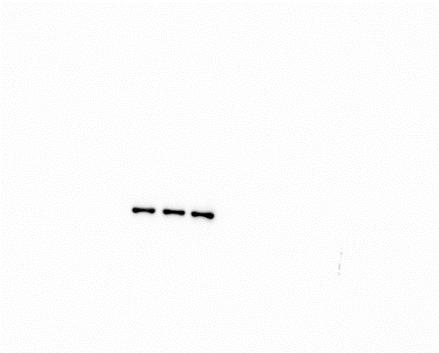

Fig. 6h

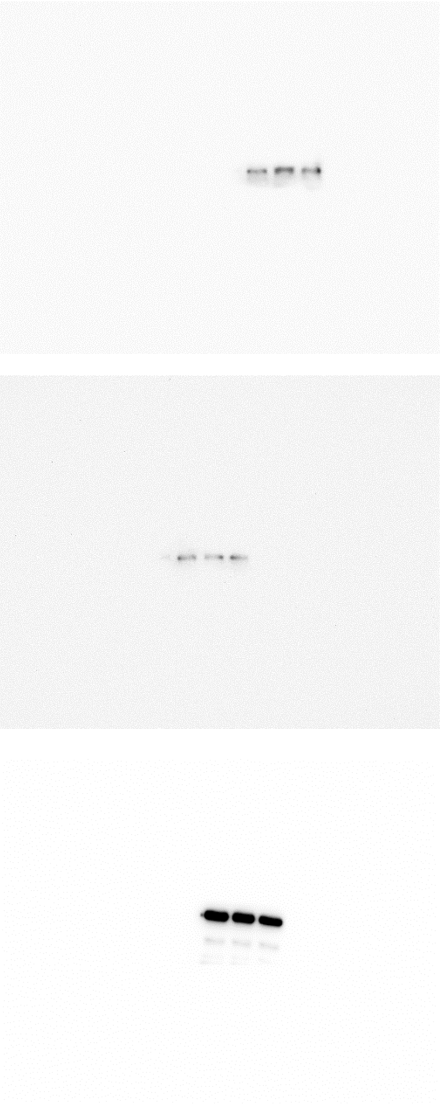

Fig. 6i

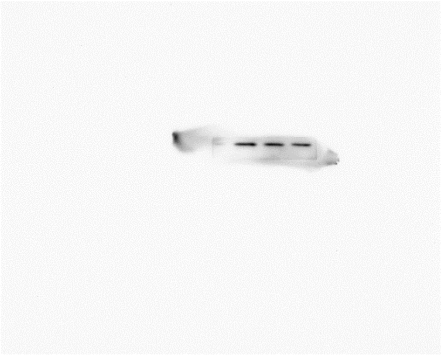

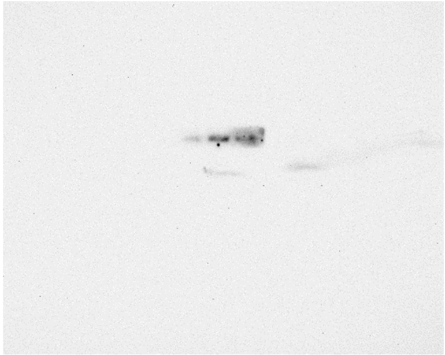

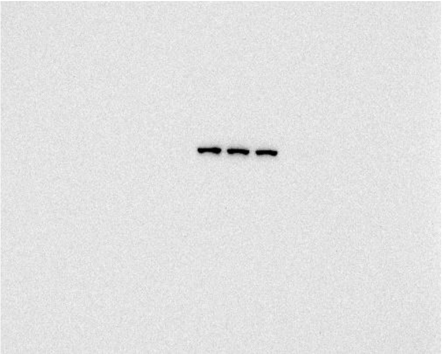

Fig. 6j

Fig. 6k
